# Effects of geographic isolation on the *Bulbophyllum* chloroplast genomes

**DOI:** 10.1186/s12870-022-03592-y

**Published:** 2022-04-19

**Authors:** Jiapeng Yang, Fuwei Zhang, Yajie Ge, Wenhui Yu, Qiqian Xue, Mengting Wang, Hongman Wang, Qingyun Xue, Wei Liu, Zhitao Niu, Xiaoyu Ding

**Affiliations:** 1 College of Life Sciences, Nanjing Normal University, Nanjing, 210023 China; 2 Jiangsu Provincial Engineering Research Center for Technical Industrialization for Dendrobiums, Nanjing, 210023 China

**Keywords:** Geographic isolation, Chloroplast genome, *Bulbophyllum*, Species evolution

## Abstract

**Background:**

Because chloroplast (cp) genome has more conserved structures than nuclear genome and mitochondrial genome, it is a useful tool in estimating the phylogenetic relationships of plants. With a series of researches for cp genomes, there have been comprehensive understandings about the cp genome features. The genus *Bulbophyllum* widely distributed in Asia, South America, Australia and other places. Therefore, it is an excellent type genus for studying the effects of geographic isolation.

**Results:**

In this study, the cp genomes of nine *Bulbophyllum* orchids were newly sequenced and assembled using the next-generation sequencing technology. Based on 19 Asian (AN) and eight South American (SA) *Bulbophyllum* orchids, the cp genome features of AN clade and SA clade were compared. Comparative analysis showed that there were considerable differences in overall cp genome features between two clades in three aspects, including basic cp genome features, SSC/IR_B_ junctions (J_SB_s) and mutational hotspots. The phylogenetic analysis and divergence time estimation results showed that the AN clade has diverged from the SA clade in the late Oligocene (21.50–30.12 mya). After estimating the occurrence rates of the insertions and deletions (InDels), we found that the change trends of cp genome structures between two clades were different under geographic isolation. Finally, we compared selective pressures on cp genes and found that long-term geographic isolation made AN and SA *Bulbophyllum* cp genes evolved variably.

**Conclusion:**

The results revealed that the overall structural characteristics of *Bulbophyllum* cp genomes diverged during the long-term geographic isolation, and the crassulacean acid metabolism (CAM) pathway may play an important role in the *Bulbophyllum* species evolution.

**Supplementary Information:**

The online version contains supplementary material available at 10.1186/s12870-022-03592-y.

## Background

Chloroplasts (cp) are semi-autonomous organelles with their own unique genomes. The cp genomes of terrestrial plants are relatively conservative, with sizes ranging from 120 to 170 kbp, and can be divided into four regions: large single copy (LSC) region, small single copy (SSC) region and two inverted repeat (IR_B_ and IR_A_) regions [1, 2]. Compared with nuclear genome and mitochondrial genome, cp genome is characterized by conserved structures, which made it a useful tool in estimating the phylogenetic relationships of plants [3, 4]. Although the cp gene content and composition are highly conserved, e.g., containing about 120 genes, mainly consisting of three categories: self-replication genes, photosynthesis genes, and RNA genes [5], the cp genomes have undergone structural changes in the long evolutionary process [6], such as IR contraction [7], inversion [8, 9], loss of genes and introns [10].

In recent years, as the sequencing costs continue to decrease, more and more complete cp genomes were sequenced and assembled, which is helpful for the plant phylogenetic studies. Orchids have become the focus of phylogenetic studies due to rich species, wide distributions and epiphytic habits. Based on 124 orchid cp genomes, Kim et al. (2020) cleared out the relationships of five subfamilies within Orchidaceae (Apostasioideae, Vanilloideae, Cypripedioideae, Orchidoideae and Epidendroideae) [4]. Moreover, there have been comprehensive understandings about the orchid cp genome features. For example, (i) cp genome substitution rates are considerably lower in IR regions than in SC regions [11, 12]; (ii) the repeat sequences played an important role in cp genome rearrangements [13–15]; (iii) mutational hotspots are genus specific [16]. However, the effects of geographic isolation on the cp genomes have not been investigated.

The genus *Bulbophyllum* Thou. belongs to the family Orchidaceae Juss., mainly distributed in Asia, South America, Australia and other places [17]. It is one of the largest genera of flowering plants, comprising about 2,000 species, which are predominantly restricted to rainforest habitats [18]. And it is characterized by epiphytic growth, CAM metabolism and fleshy flowers. There are about 105 species in China, which are widely distributed in Guizhou, Guangxi, Yunnan and Fujian provinces [19]. The wide distributions make the genus *Bulbophyllum* an excellent mode for studying the effects of geographic isolation on the cp genomes. There have been previous studies on the *Bulbophyllum* phylogenetic relationships. Gamisch et al. (2015) used internal transcribed spacer (ITS) sequences and four short cp genome fragments to study the phylogenetic relationships and estimate divergence times of Madagascar *Bulbophyllum* [20]. Gamisch et al. (2019) analyzed the evolutionary relationships of *Bulbophyllum* between different continents with ITS sequences, showing that Asian (AN) clade first separated from other *Bulbophyllum* orchids, and then South American (SA) clade separated from African *Bulbophyllum* [18]. Therefore, the *Bulbophyllum* is an excellent type genus to study the effects of geographical isolation on cp genomes. 

In this study, the cp genomes of nine AN *Bulbophyllum* orchids were newly sequenced and assembled using the next-generation sequencing technology. The comprehensive comparative analyses were performed, based on 27 complete cp genomes of AN and SA *Bulbophyllum*. The aims of this study were: (1) to uncover the differences in cp genome features between AN clade and SA clade; (2) to assess the effects of long-term geographic isolation on cp genome structures; (3) to investigate the direct causes of the variable selective pressures on cp genes. We believed that this study will give us a comprehensive understanding of the effects of geographic isolation on the complete cp genomes.

## Results

### Chloroplast genome features

**Fig. 1:**
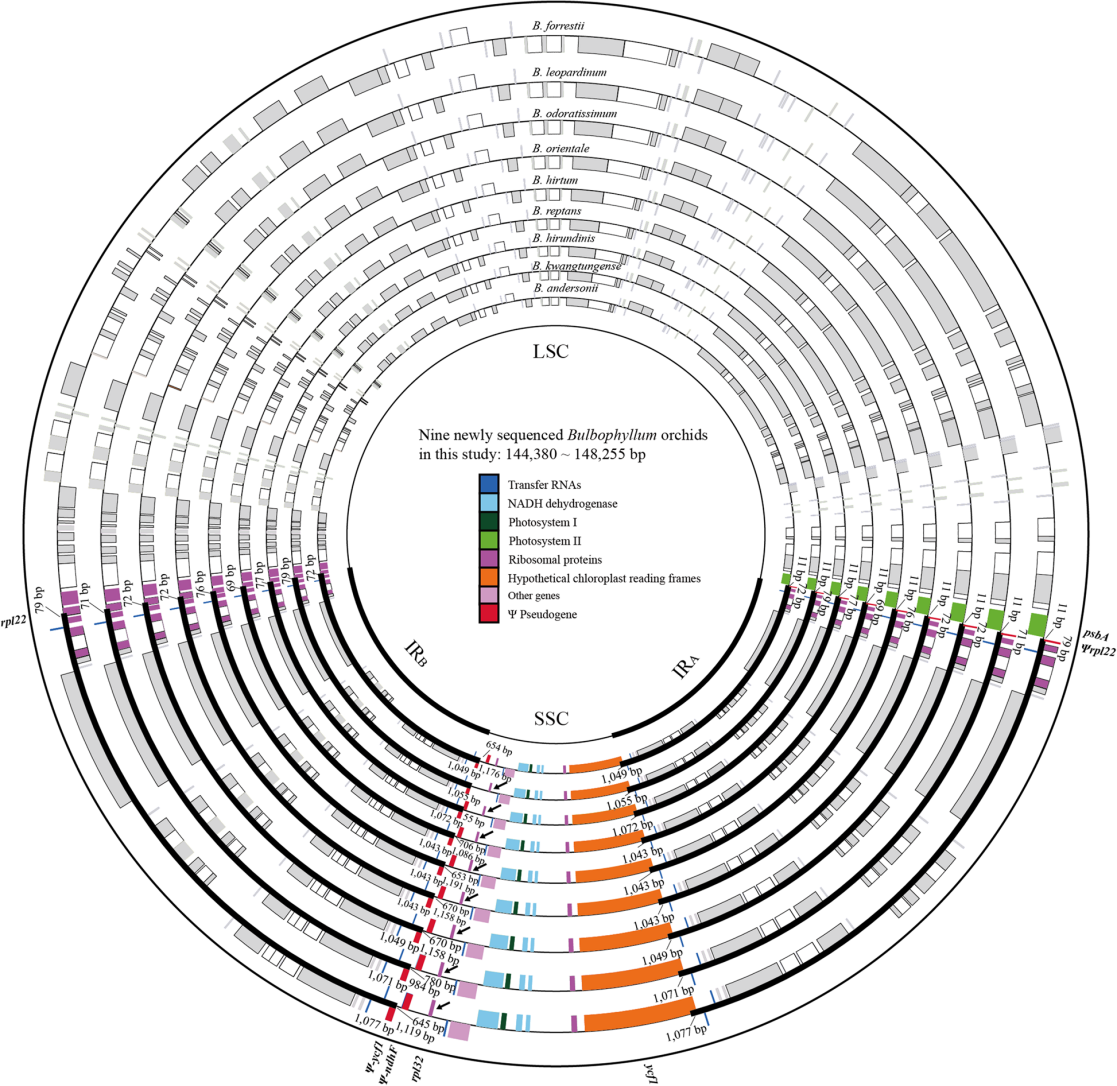
Cp genomes map of *Bulbophyllum* orchids. The lengths of the cp genomes are shown in the circle. Cp genes at four junctions and SSC regions are colored in

The nine newly sequenced *Bulbophyllum* cp genomes were circular with the typical quadripartite structure, including a large single copy (LSC), a small single copy (SSC) and a pair of inverted repeats (i.e., IR_A_ and IR_B_) (Fig. 1). We combined the published cp genomes of ten AN and eight SA *Bulbophyllum* orchids with this study’s nine AN *Bulbophyllum* orchids to compare the basic cp genome features of two clades. As shown in Table 1, the AN cp genome sizes were ranged from 144,380 bp to 158,524 bp, with GC contents of 36.66–38.04%. The cp genomes were variable in LSC and SSC regions, with 77,088 to 86,200 bp and 12,140 to 18,632 bp, while conserved in IR regions, with sizes ranging from 25,824 to 26,919 bp. The AN cp genome GC contents varied slightly in LSC (34.20–36.05%), SSC (29.00–30.93%) and IR (43.03–43.38%) regions. Notably, except for IR regions, the GC contents of AN clade were higher than SA clade (Mann–Whitney two-sided, *P* < 0.05) [17], which indicated that there were significant cp genome differences between two clades. Moreover, the *Bulbophyllum* cp genomes were well-conserved in gene number and gene order, with 74 protein-coding genes, 30 tRNA genes and 4 rRNA genes.

**Table 1:** The features of *Bulbophyllum* cp genomes

No	Species	Accession	Reference	Cp genome	LSC	IR	SSC	GC content (%)			
		no		length (bp)	length (bp)	length (bp)	length (bp)	Total	LSC	IR	SSC
1	*B. disciflorum*	LC498826	Tang et al. (2019) [21]	148,554	79,001	26,378	16,797	37.94	35.80	43.38	30.93
2	*B. gedangense*	MW161053	Tang et al. (2021) [22]	158,524	86,200	26,846	18,632	36.76	34.47	43.05	29.26
3	*B. lingii*	MW161052	Tang et al. (2021) [22]	156,669	84,607	26,919	18,224	36.77	34.35	43.09	29.30
4	*B. menghaiense*	MW161050	Tang et al. (2021) [22]	156,550	84,663	26,891	18,105	36.66	34.20	43.03	29.26
5	*B. pentaneurum*	MW161051	Tang et al. (2021) [22]	156,142	84,240	26,838	18,226	36.81	34.44	43.05	29.40
6	*B. pingnanense*	MW822749	Zhang et al. (2021) [23]	151,224	86,017	25,855	13,497	36.98	34.45	43.21	29.25
7	*B. inconspicuum*	NC_046811	Kim et al. (2020) [4]	149,548	85,760	25,824	12,140	37.00	34.41	43.17	29.00
8	*B. forrestii*	LC642719	This study	148,067	78,167	26,414	17,072	37.91	35.86	43.29	30.63
9	*B. hirtum*	LC642724	This study	147,382	77,587	26,333	17,129	37.96	35.93	43.34	30.60
10	*B. hirundinis*	LC642721	This study	144,380	77,399	26,437	14,107	38.02	35.79	43.22	30.76
11	*B. kwangtungense*	LC642722	This study	145,092	77,192	26,262	15,376	37.98	35.93	43.31	30.03
12	*B. leopardinum*	LC642723	This study	147,514	77,762	26,378	16,996	38.04	36.05	43.37	30.58
13	*B. odoratissimum*	LC642720	This study	147,494	77,664	26,290	17,250	37.88	35.87	43.32	30.34
14	*B. orientale*	LC642725	This study	147,388	77,392	26,395	17,206	38.01	36.05	43.29	30.65
15	*B. reptans*	LC642726	This study	146,928	77,088	26,401	17,038	37.98	35.98	43.29	30.58
16	*B. andersonii*	LC703293	This study	148,255	78,074	26,366	17,449	37.83	35.84	43.32	30.19
17	*B. affine*	LC556091	Yang et al. (2020) [24]	148,230	78,178	26,386	17,280	37.86	35.83	43.30	30.38
18	*B. pectinatum*	LC556092	Yang et al. (2020) [24]	147,169	77,478	26,081	17,529	38.01	36.01	43.37	30.87
19	*B. funingense*	LC556093	Yang et al. (2020) [24]	147,464	77,564	26,403	17,094	37.92	35.89	43.25	30.68
20	*B. epiphytum*	NC_048486	Zavala-Páez et al. (2020) [17]	147,546	82,354	25,847	13,498	36.73	34.06	43.20	28.20
21	*B. exaltatum*	NC_048480	Zavala-Páez et al. (2020) [17]	150,410	83,335	25,847	15,381	36.78	34.21	43.22	29.09
22	*B. granulosum*	NC_048481	Zavala-Páez et al. (2020) [17]	151,112	84,492	25,465	15,690	36.72	34.26	43.13	29.13
23	*B. mentosum*	NC_048482	Zavala-Páez et al. (2020) [17]	150,217	83,642	26,338	13,899	36.74	34.14	43.11	28.28
24	*B. plumosum*	NC_048479	Zavala-Páez et al. (2020) [17]	146,401	83,260	26,025	11,091	36.56	33.72	43.00	27.62
25	*B. regnellii*	NC_048483	Zavala-Páez et al. (2020) [17]	151,493	84,868	25,542	15,541	36.66	34.17	43.09	29.11
26	*B. steyermarkii*	NC_048484	Zavala-Páez et al. (2020) [17]	146,720	83,488	25,953	11,326	36.74	34.20	42.80	27.72
27	*B. weddellii*	NC_048485	Zavala-Páez et al. (2020) [17]	151,355	83,450	25,927	16,051	36.64	34.09	43.16	28.90

The levels of the nine newly sequenced cp genome sequence variability were estimated and plotted using mVISTA, with *Dendrobium huoshanense* as the reference (Fig. S1). As expected, the non-coding regions (intergenic spacers and introns) were more divergent than coding regions, and the SC regions exhibited higher divergence levels than IR regions. Comparative cp genomic analysis of *Bulbophyllum* revealed a minor organizational variation, which covered an inversion of *rpl32*.

### Repeat and SSR analysis

The Longer dispersed repeats (LDRs) were detected using REPuter and shown in Fig. S2A. The results showed that, (i) the LDRs distribution densities in SSC region were higher than LSC and IR regions (Mann–Whitney two-sided, *P* < 0.05). (ii) The LDRs in SSC regions were highly varied between two clades (T-test for Independent Samples: *P* < 0.05). The analysis of simple sequence repeats (SSRs) also revealed the identical trends (Fig. S2B). Considering the higher LDRs and SSRs distribution densities with the lower GC contents in SSC regions, we conducted correlation tests to see if there were correlative relationships among them. Indeed, significant negative correlations were detected among them (LDRs vs GC contents: Pearson's *r* = -0.529; SSRs vs GC contents: Pearson's *r* = -0.977, *P* < 0.05), which indicated that low GC contents may lead to the SSC regions more variable.

### Comparison of sequences flanking IR/SC junctions between Asian and South American clades

The sequences flanking IR/SC junctions in cp genomes were compared. As shown in Fig. 1 and Fig. S3, the *Bulbophyllum* IR/SC junctions were highly conserved. The SSC/IR_A_ junctions (J_SA_s) was located in the 5’ end of *ycf1*, which resulted in a pseudogene fragment *Ψycf1* (33 to 1,434 bp) in the SSC/IR_B_ junctions (J_SB_s). Meanwhile, the LSC/IR_B_ junctions (J_LB_s) were located in *rpl22*, which leaded *Ψrpl22* (10 to 79 bp) in the LSC/IR_A_ junctions (J_LA_s). Remarkably, IR_B_ expanded progressively from J_SB_s to *ndhF*. The main differences between the two clades were in J_SB_, which can be divided into three types, (A) a relatively long *Ψycf1* (796 to 1,434 bp) with a *ΨndhF* (*B. forrestii*, *B. leopardinum*, *B. odoratissimum*, *B. orientale*, *B. hirtum*, *B. hirundinis*, *B. reptans*, *B. menghaiense*, *B. andersonii*, *B. affine*, *B. pectinatum*, *B. funingense*, *B. pentaneurum*, *B. disciflorum*, *B. gedangense*, *B. lingii*, *B. steyermarkii*); (B) a short *Ψycf1* (156 to 360 bp) with a *ΨndhF*, including *B. mentosum* and *B. epiphytum*; (C) no *ΨndhF*, including *B. kwangtungense*, *B. inconspicuum*, *B. pingnanense*, *B. regnellii*, *B. exaltatum*, *B. granulosum*, *B. plumosum* and *B. weddellii*. However, there was no type B in AN clade. Meanwhile, there were more type C in SA clade. These results revealed that the evolution of IR/SC junctions between two clades were diversified.

### Diverse mutational hotspots between the Asian and South American clades

**Fig. 2:**
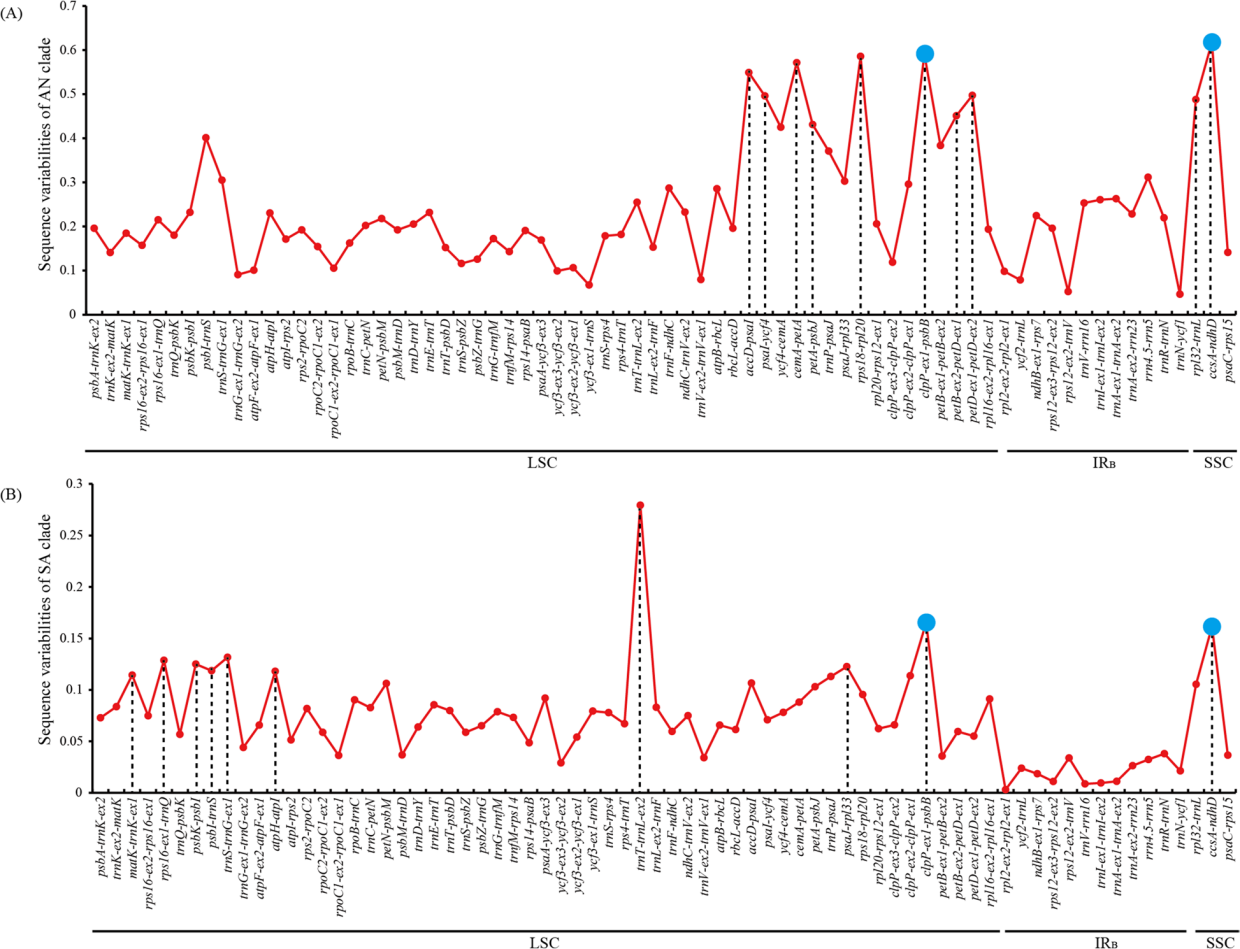
Sequence variabilities of non-coding loci among AN and SA clades. Dashed lines indicate top ten hotspots. Blue points indicate common top ten hotspots of two clades

We extracted a total of 72 intergenic and intronic loci (conserved sites > 100 bp) from two clades and calculated their sequence variabilities (SVs), with *D. huoshanense* as the reference. As shown in Fig. 2, the SVs in SC regions were significantly higher than IR regions (Mann–Whitney two-sided, *P* < 0.05). In the AN clade, the top-ten loci (SV = 0.4309 to 0.6177) mainly located in LSC region (eight in LSC and two in SSC) (Fig. 2A). And in the SA clade, only two mutational hotspots were identical with the AN clade (*ccsA-ndhD* and *clpP-ex1-psbB*) (Fig. 2B), which suggested that the evolution of non-coding regions between two clades were disproportional.

### Geographic isolation verification

The aligned cp genomes and ITS sequences were connected to test hybridization. The ten AN *Bulbophyllum* orchids were set as the BAN branch, the seven SA *Bulbophyllum* orchids were set as the BSO branch, two *D. huoshanense* species were set as the DHU branch, four *D. moniliforme* species were set as the DMO branch and five *D. officinale* species were set as the DOF branch (Fig. S4). The JML results showed that no hybridization occurred between BAN branch and BSO branch. These results proved that there was no gene flow between two clades, which may due to the long periods of geographic isolation.

### Phylogenetic analysis and divergence time estimation

**Fig. 3:**
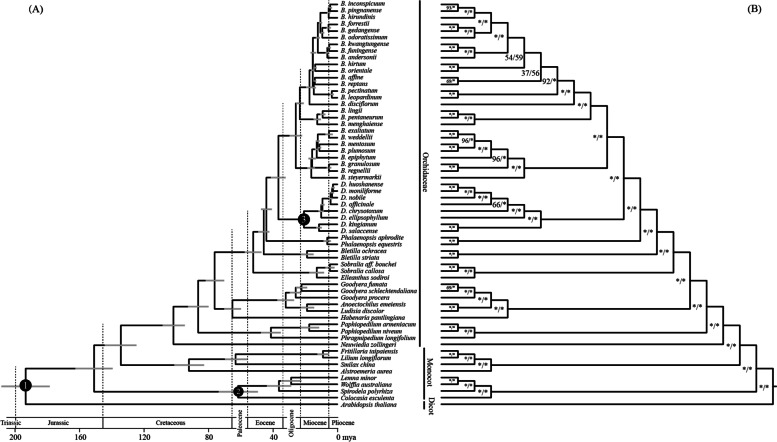
Chronogram and Phylogenetic relationships of 61 angiosperms. **a** Molecular dating results of 61 angiosperms by BEAST2; **b** ML tree topology with ML and BI bootstrap values. The first one represents ML bootstrap value and the second one represents BI bootstrap value. * represents 100 bootstrap value

The phylogenetic relationships among 27 *Bulbophyllum* species were reconstructed by 61 angiosperm cp genomes (Table S1). The ML tree exhibited identical topology with the BI tree. As shown in the Fig. 3B, the phylogenetic trees revealed a monophyletic relationship for the orchids with strongly support (bootstrap values = 100/100). Meanwhile, the *Bulbophyllum* achieved a monophyly with 100/100 bootstrap values, and was sister to *Dendrobium*. As expected, the 27 *Bulbophyllum* orchids were divided into the AN clade and SA clade. These results were consistent with previous studies [4, 18, 25].

ITS sequences and three cp genes (*psbA*, *matK* and *rbcL*) were used to reconstructed the phylogenetic relationships of 27 orchids (including *Bulbophyllum*, *Dendrobium* and *Phalaenopsis*) (Fig. S5). The results supported that 27 *Bulbophyllum* orchids were strictly divided into two clades. In addition, we found that neither the ITS tree nor the ITS + *psbA* + *matK* + *rbcL* tree was comparable to cp genome tree in bootstrap values. Obviously, the cp genomes have a better performance in studying plant phylogenetic relationships.

The divergence times of 61 orchids were estimated using BEAST2. The topologies of the BEAST tree were almost consistent to ML and BI trees except for *B. affine* and *B. reptans*, which may be because reconstructing phylogenetic relationships at the angiosperm-scale hid SNPs that can distinguish between close species (Fig. 3A). In this study, *Bulbophyllum* was inferred to have diverged from *Dendrobium* at 36.77 (32.15–41.39) million years ago (mya), and the *Bulbophyllum* AN clade was inferred to have diverged from SA clade at 25.81 (21.5–30.12) mya. Notably, these results were slightly higher than previous studies [18, 25], mainly because cp genome sequences are longer than ITS sequences and other short fragments.

### Cp genome structural variation analysis

**Fig. 4:**
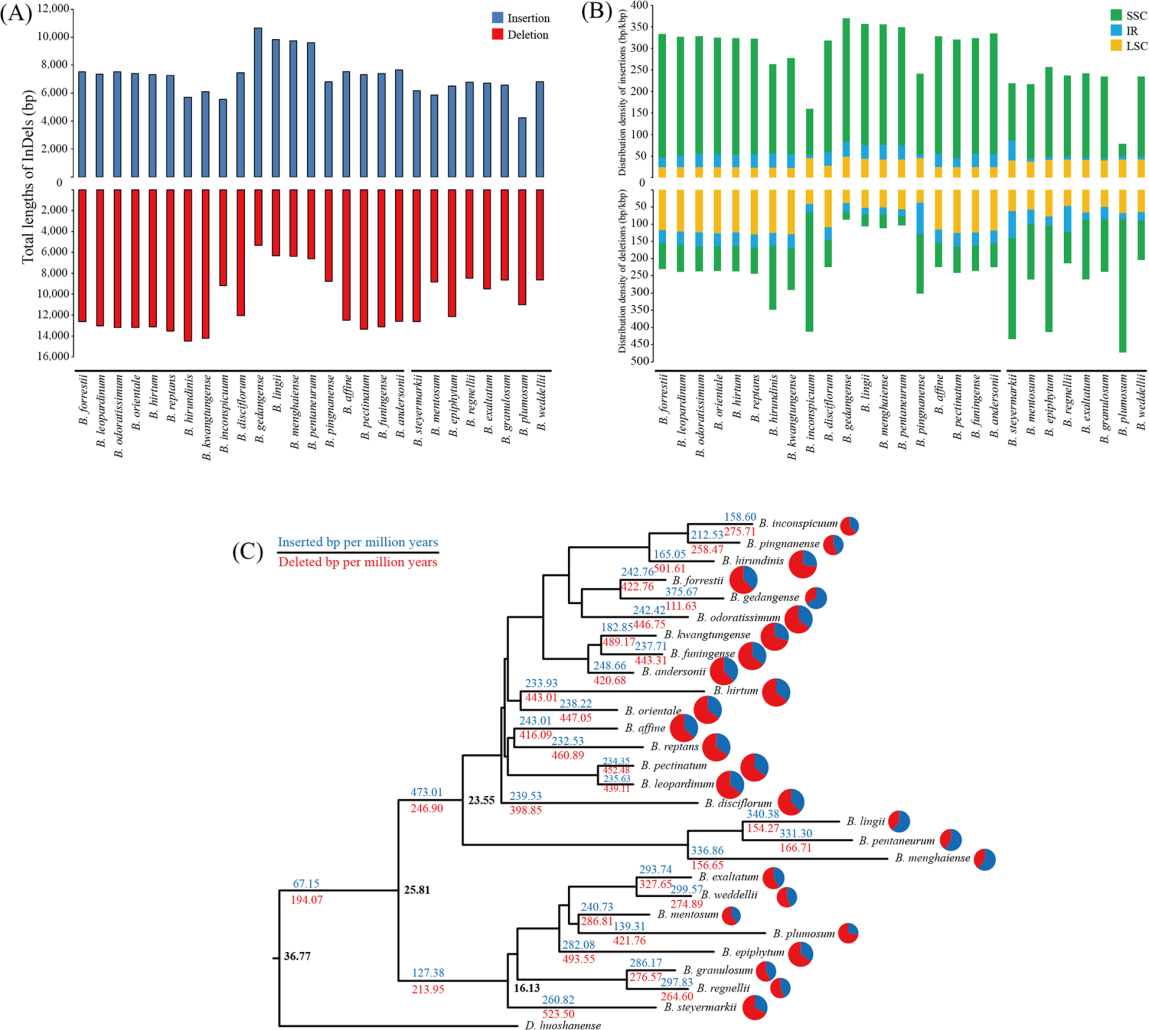
InDels analysis results of *Bulbophyllum* orchids. **a** Total InDels lengths with reference *D. huoshanense*; **b** InDels distribution densities in different regions of *Bulbophyllum* orchids; **c** InDels occurrence rates of different branches in *Bulbophyllum*

The insertions and deletions (InDels) were identified in 27 *Bulbophyllum* cp genomes, with *D. huoshanense* as outgroup. There were more deletions (5,314 to 14,498 bp) than insertions (4,216 to 10,652 bp) in *Bulbophyllum* (Fig. 4A). In addition, the distribution of InDels were different between AN and SA clades. For example, (i) the InDels lengths were varied between two clades. The total InDels lengths of AN clade (5,540 to 10,652 bp and 5,314 to 14,498 bp) were higher than SA clade (4,216 to 6,801 bp and 8,466 to 12,642 bp) (Fig. 4A). (ii) Except for partial AN *Bulbophyllum* species, the InDels distribution densities in SSC region were higher than in LSC and IR regions (Fig. 4B), which indicated that SSC region structure was diversified among two clades. (iii) In addition to InDels distribution densities in different regions, the occurrence rates of InDels between AN and SA clades were also inconsistent (Fig. 4C). The InDels occurrence rates of AN clade (473.01 and 246.90 bp/myr) were higher than SA clade (127.38 and 213.95 bp/myr). These results revealed that the evolution of cp genome structures between two clades were asymmetry.

### Evolution analysis of protein-coding genes

**Fig. 5:**
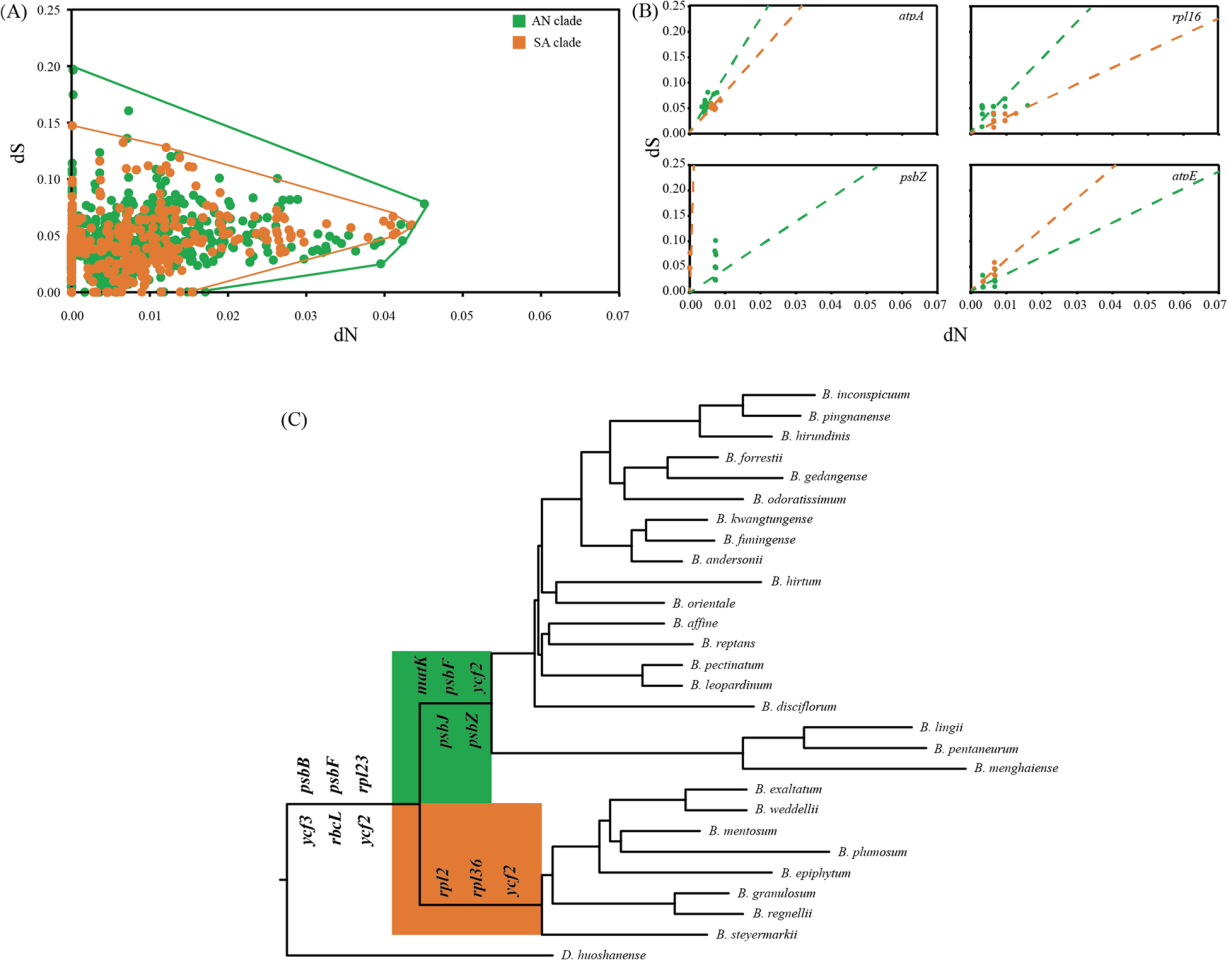
Selective pressure analysis results in *Bulbophyllum*. **a** Scatter diagram for dN and dS of 63 cp protein-coding genes from 27 *Bulbophyllum* orchids with reference *D. huoshanense*; **b** Scatter diagram for dN and dS of *atpA*, *psbZ*, *rpl16* and *atpE*; **c** ML tree with positively selective genes in *Bulbophyllum* branch, AN branch and SA branch, respectively

The synonymous (dS) and non-synonymous (dN) substitution rates for each protein-coding genes were estimated using codonML, with *D. huoshanense* as reference. The values of dS (0–0.1973) were higher than dN (0–0.0451) in *Bulbophyllum*. Although most of ω values between two clades were similar (Fig. 5A), there were some significantly different protein-coding genes, such as *atpA*, *psbZ*, *rpl16* and *atpE* (Fig. 5B, Table S2). These genes, which mainly contained photosynthesis and self-replication functions, were mostly distributed in SC regions (14 in SC regions, two in IR regions) (Table S3). These results indicated that a part of protein-coding genes between two clades were under different selective pressures.

The Branch model of codeML were used to investigate the possible role of positive selection in driving protein-coding gene evolution among three branches (*Bulbophyllum* branch, AN branch and SA branch). The comparison showed that a part of genes was under different selective pressures. For example, there were six genes (*psbB*, *psbF*, *rbcL*, *rpl23*, *ycf3* and *ycf2*) were found with positive selection in the *Bulbophyllum* branch; and five genes (*matK*, *psbJ*, *ycf2*, *psbZ* and *psbF*) were found in the AN branch; and three genes (*rpl2*, *rpl36* and *ycf2*) were found in the SA branch (Fig. 5C). Most of them were in the LSC region (Table S4). The positively selective protein-coding genes between AN and SA branches were different, which suggested that different environments diversified the evolution of the protein-coding genes.

## Discussion

### Chloroplast genomes of *Bulbophyllum* Asian and South American clades were different

Comparative cp genome analyses have revealed that cp genomes are conserved in genome sizes, structures, and gene contents [26–28]. Because evolutionary rates of cp genomes are slower than nuclear and mitochondrial genomes [3, 29], complete cp genomes are useful resources for phylogenetic analysis and species identification [30–32]. With the rapid development of cp genome researches, there have been comprehensive understandings about the cp genome characteristics. For example, (i) cp genome substitution rates are considerably lower in IR regions than in SC regions [3, 11, 12]; (ii) the repeat sequences played an important role in rearrangements of cp genomes [13–15]; (iii) mutational hotspots are genus specific [33, 34]. However, the differences of the cp genomes in different continents have not been analyzed. The genus *Bulbophyllum*, which is widely distributed in Asia, south America and other places [17, 18], is an excellent source for the comparison of cp genome features, cp genome junctions and mutational hotspots.

We compared the overall structures and variations of *Bulbophyllum* cp genomes. All results showed that there were considerable differences in overall cp genome features between AN and SA clades. Firstly, they have different cp genome features. For example, (i) the overall, LSC and SSC GC contents of AN clade are higher than SA clade; (ii) part of the *rpl32* orientations in AN clade were different from SA clade, which caused by an inversion; (iii) except LSC LDRs and IR SSRs distribution densities, the LDRs and SSRs distribution densities in SA clade were higher than in AN clade. Secondly, J_SB_s between two clades varied significantly. AN and SA clades had different types of J_SB_s. There was no type B J_SB_s in AN clade and there were more type C J_SB_s in SA clade. Thirdly, the distributions and kinds of mutational hotspots between two clades were significantly different. Niu et al. (2017) found specific barcodes searched by 8–10 closely related cp genomes from specific genus can uncover their cp genome features [33]. Therefore, the mutational hotspots of two clades estimated in this study can reflect the variations of non-coding loci. However, there were only two hotspots shared between two clades, which indicated that the AN and SA clades had discordant evolution in non-coding regions. In conclusion, AN and SA clades had considerable differences in overall cp genome features.

### The cp genome structures between Asian and South American clades were different under geographic isolation

Hybridization, a driver of plant evolution, is common among terrestrial orchids [35]. Although dust-like seeds have the capacity to travel long distance [36–38], the results of this study showed that *Bulbophyllum* AN clade has diverged completely from SA clade in the late Oligocene (21.50–30.12 mya) [18]. Because the results of the JML software have ruled out the possibility of hybridization between the two clades [39], *Bulbophyllum* AN and SA clades have evolved independently for at least 25.81 million years. Our subsequent analyses revealed significant cp genome structural variations between two clades.

The insertions and deletions (InDels) are important structural variations of cp genomes [32]. In order to evaluate the structural variations of the cp genomes during the evolution of *Bulbophyllum*, we calculated the total InDels lengths of each *Bulbophyllum* by the aligned complete cp genome sequences, with *D. huoshanense* as outgroup. There were 5,314 to 14,498 bp deletions and 4,216 to 10,652 bp insertions in *Bulbophyllum*. Obviously, the lengths of deletions were longer than insertions, which is consistent with previous studies [16, 40]. To obtain more detailed information about InDels, we calculated the distribution densities of InDels in different cp genome regions. The distribution densities of insertions in LSC, IR, and SSC regions were 22.7–48.9 bp/kbp, 4.8–46.1 bp/kbp, and 29.4–286.9 bp/kbp, respectively. And the deletions distribution densities in LSC, IR and SSC regions were 37.8–130.5 bp/kbp, 16.2–92.3 bp/kbp, and 24–389.1 bp/kbp, respectively. The results revealed that the distributions of InDels are dependent on their locations in cp genomes [16, 41, 42], e.g., associated with low GC contents.

To estimate the pace of structural variations in the *Bulbophyllum* cp genomes, we dated the occurrence rates of InDels by located them at the BEAST tree branches. The results showed that, during the past ~ 10.96 million years since the late Eocene, the insertions and deletions occurred at the speeds of 67.15 bp/myr and 194.07 bp/myr. Then, AN clade and SA clade diverged at ~ 25.81 mya. Our results further revealed that InDels remarkably varied across different lineages [40]. Nevertheless, the most InDels occurrence rates of AN *Bulbophyllum* were higher than SA *Bulbophyllum*. We speculated that there were two main reasons for the higher occurrence rates of InDels in the AN clade: (1) the results of Gamisch et al. (2021) indicated that Asia was the original continent of the *Bulbophyllum* orchids [43]; (2) Of all the continents where *Bulbophyllum* orchids grow, Asia has the largest suitable area for speciation and niche expansion of the genus *Bulbophyllum* [43]. In conclusion, the cp genome structures between two clades were different under geographic isolation. And for further study, InDels may be an important indicator of species evolution.

### Crassulacean acid metabolism (CAM) pathway may be the inducement of cp gene differentiation

Crassulacean acid metabolism (CAM) is the typical plant photosynthetic pathway that improves water use efficiency of plants [44]. About 10% of orchid species have been confirmed to be CAM plants [45–47], and 50% of Orchidaceae species were anticipated to be CAM plants [48]. CAM has evolved multiple independent times in the Orchidaceae [49]. Phylogenetic analyses of the neotropical orchids indicated that CAM has evolved independently at least 10 times [48]. For example, CAM in *Dendrobium*, the sister genus of *Bulbophyllum*, has arisen for at least eight times [49]. The *Bulbophyllum* orchids are typical CAM orchids. Although the C_3_ pathway is the main photosynthetic pathway of *Bulbophyllum* presently, the CAM pathway plays an important role in adapting to new ecological niches and increasing species diversity [43].

In order to evaluate the effects of CAM pathway on cp gene evolution in the genus *Bulbophyllum*, Selective pressure analysis of functional genes has been employed [50–52]. Non-synonymous (dN), synonymous (dS) nucleotide substitution rates and non-synonymous/synonymous rate ratio (ω = dN/dS) are widely used as indicators of adaptive evolution [40]. The values of dN and dS on all protein-coding genes between two clades were compared, and we found three photosynthesis genes, ten self-replication genes and three other genes, which have significantly different ω values. The three photosynthesis genes (*psbZ*, *atpE* and *atpA*) encode fractions of the photosystems and the thylakoid ATP synthase complex. In addition, positively selective genes (ω values > 1) through the *Bulbophyllum* evolution were screened. There were six, five and three positively selective cp genes in the *Bulbophyllum* branch, AN branch and SA branch, respectively. And these genes were mostly distributed in the LSC regions, which is consistent with previous studies [40]. Because there were many photosynthesis genes, which are components of Photosystem II, among two clades under different selective pressures, the CAM pathway may play an important role in adaptive evolution of *Bulbophyllum*. In addition, Hu et al. (2022) assessed the origin and loss of CAM in the *Bulbophyllum* since middle Miocene using two cp genes (*matK* and *psbA*) and two nuclear sequences (ITS and *Xdh*) [53]. They found that although CAM increased the adaptability of the *Bulbophyllum* plants to specific conditions, it was less competitive than C_3_ in the long term. Predictably, the role of CAM in the species evolution of *Bulbophyllum* can be further explored using the complete cp genomes.

Before the ancestors of the *Bulbophyllum* diverged from *Dendrobium* at late Eocene, CAM and epiphytism in orchids are likely to have originated about 65 mya [54, 55]. Then, some of the *Bulbophyllum* photosynthetic genes (*psbB*, *psbF* and *rbcL*) had evolved to adapt to the changeable environment and expand habitat region. At late Oligocene, AN clade diverged from other *Bulbophyllum* clade, through which SA clade had no gene flow with AN clade. The cp genomes of two clades evolved to different features under different environments. In this process, the CAM pathway affects the selective pressures of photosynthetic genes. Therefore, in subsequent studies, CAM studies and cp genome studies will be more closely linked to explore species evolution of *Bulbophyllum*.

## Conclusions

In this study, the nine *Bulbophyllum* cp genomes were newly sequenced and assembled. Comparative analyses showed that there were considerable differences in overall cp genome features between AN and SA clades in three aspects, including basic cp genome features, JS_B_s and mutational hotspots. Then we found that the cp genome structures between two clades were different under geographic isolation by estimating the occurrence rates of the InDels. Finally, the results showed that long-term geographic isolation put AN and SA *Bulbophyllum* cp genes under different selective pressures. All results indicated that the overall structural characteristics of *Bulbophyllum* cp genomes diverged during the long-term geographic isolation, and the CAM pathway may play an important role in the cp genes evolution of *Bulbophyllum*.

## Methods

### Plant materials and DNA extraction

In this study, nine medicinal *Bulbophyllum* orchids sequenced (*B. forrestii* Seidenf. (voucher specimen: Yang202101), *B. leopardinum* (Wall.) Lindl. (voucher specimen: Yang202102), *B. odoratissimum* Lindl. (voucher specimen: Yang202103), *B. orientale* Seidenf. (voucher specimen: Yang202104), *B. hirtum* (J. E. Smith) Lindl. (voucher specimen: Yang202105), *B. reptans* Lindl. (voucher specimen: Yang202106), *B. kwangtungense* Schltr. (voucher specimen: Yang202107), *B. hirundinis* (Gagnep.) Seidenf. (voucher specimen: Yang202108) and *B. andersonii* (Hook. f.) J. J. Smith (voucher specimen: Yang202201)) were collected in Yunnan and Fujian provinces, China, and stored in College of Life Sciences, Nanjing Normal University, Nanjing, China. All the samples were identified by Prof. Dr. Xiaoyu Ding. Total genomic DNA of each sample was extracted from 0.2 g fresh leaves using Dneasy Plant Mini Kits (QIAGEN, Germany). DNA samples that met the quality requirements (A260/280 ratio = 1.8–2.0, A260/230 ratio > 1.7, and DNA concentration > 300 ng/μL) were used for sequencing. In addition, ten published AN *Bulbophyllum* (*B. disciflorum*, *B. gedangense*, *B. lingii*, *B. menghaiense*, *B. pentaneurum*, *B. pingnanense*, *B. inconspicuum*, *B. affine*, *B*. *pectinatum and B*. *funingense*) and eight SA *Bulbophyllum* (*B. epiphytum*, *B. exaltatum*, *B. granulosum*, *B. mentosum*, *B. plumosum*, *B. regnellii*, *B. steyermarkii* and *B. weddellii*) cp genomes were selected for subsequent comparative genome analysis.

### DNA sequencing, assembly and annotation

The total genomic DNA of nine *Bulbophyllum* orchids was sequenced using Illumina Hiseq4000 platform (Illumina Inc, San Diego, CA, USA). Approximately 5.0 Gb of raw data were generated with 150 bp paired-end reads for each sample. The fragments with coverage < 50 × were deleted and filtered paired-end reads were assembled on CLC Genomics Workbench v8.5.1 (CLC Bio, Aarhus, Denmark), with reference *Dendrobium huoshanense* C. Z. Tang et S. J. Cheng. The four junctions between LSC/SSC and IRs were validated with PCR amplification. The assembled cp genomes were annotated using DOGMA v1.2 and tRNAscan-SE v1.21 [56, 57]. The boundaries of annotated genes were confirmed by multiple sequence alignment.

### Comparative analysis of cp genomes

The gene location information was extracted, and the IR/SC junction information was collated with the gene location information. The GC contents of 27 *Bulbophyllum* orchids were analyzed. The nine newly sequenced *Bulbophyllum* cp genomes were compared using online mVISTA (https://genome.lbl.gov/vista/mvista/submit.shtml) on LAGAN model, with reference *D. huoshanense* [58].

### Characterization of repeat sequences and simple sequence repeats

The REPuter program (https://bibiserv.cebitec.uni-bielefeld.de/reputer) was used to identify the four different types of repeats (forward, reverse, complement and palindromic repeats), with sequence identity ≥ 90%, minimum repeat size ≥ 30 bp, and Hamming distance = 3 [59]. Simple sequence repeats (SSRs) were discovered using the online program MISA (https://webblast.ipk-gatersleben.de/misa/) with the following parameters: 8 for mononucleotide motifs, 5 for polynucleotide motifs [60]. Then the distribution densities of repeat sequences and SSRs on LSC, SSC and IR regions were compared.

### IR/SC junctions map

Refer to the method of Zhu et al. (2018) [42], the IR/SC junctions map of 19 AN *Bulbophyllum* orchids, eight SA *Bulbophyllum* orchids and *D. huoshanense* was drawn.

### Mutational hotspots

Based on 19 AN *Bulbophyllum* cp genomes, the top ten hotspots in AN clade were screened. Based on eight SA *Bulbophyllum* cp genomes, the top ten mutational hotspots in SA clade were screened. The gene location information was transformed into the non-coding region location information, and the sequence of each non-coding region was extracted (*ycf1-rpl32* was taken as a whole fragment due to the loss of *ndhF*). Each non-coding region was aligned with MEGA 5.0 [61], and gaps at both ends were deleted. SNPs, InDels, and conserved sites were calculated by DnaSP v5 [62]. Non-coding regions, of which conserved sites were less than 100 bp, were removed. Sequence variabilities were calculated as follows: Sequence Variabilities = (SNPs + InDels) / (SNPs + InDels + Conserved sites) × 100% [34, 63].

### Hybridization testing

The cp genomes and ITS sequences of 17 *Bulbophyllum* orchids, two *D. huoshanense*, four *D. moniliforme* and five *D. officinale* were aligned using MAFFT 7.220 [64], and were connected together. Under the rule of AIC (Akaike information criterion), the optimum base substitution model calculated by Modeltest 3.7 was GTR + I + Γ [65]. The species tree was constructed using BEAST2 [66]. The Markov Chain Monte Carlo (MCMC) chains were performed to check convergence with 10,000,000 generations. The probability of hybridization was calculated using JML [67]. The significance level was 0.01.

### Phylogenetic analysis

Phylogenetic relationships were analyzed based on 61 complete cp genomes, including one dicotyledonous plant, eight monocotyledonous non-orchids, and 52 orchids (Table S1). The cp genome sequences were aligned using MAFFT 7.220 [64]. The conserved loci were selected by Gblocks v.0.91b with the following parameters: minimum number of sequences for a conserved position: 37, minimum number of sequences for a flank position: 62, maximum number of contiguous non-conserved positions: 8, minimum length of a block: 10, allowed gap positions: none [68]. Under the rule of AIC (Akaike information criterion), the optimum base substitution model calculated by Modeltest 3.7 was GTR + I + Γ [65]. The Maximum Likelihood (ML) phylogenetic tree was constructed using RAxML (version 7.4.2) with 1000 rapid bootstrap inferences [69]. The outgroup was *Arabidopsis thaliana*. The BI analysis was made by using MrBayes 3.2.7 with 1000,000 generations [70]. Trees were sampled every 1,000 generations, and the first 25% of these were discarded. The remaining trees were used to build the Bayesian tree of posterior probabilities. In addition, ITS, *psbA*, *matK*, *rbcL* and cp genomes of 27 orchids were used to reconstructed ML trees with 1000 rapid bootstrap inferences.

### Divergence time estimation

Divergence times were estimated by BEAST2 [66]. The following constraints were used for time calibrations: (1) The root age was set as 194 million years ago (Mya) (prior distribution: normal, mean: 194, sd: 8) [71–73]. (2) The stem age of Lemnoideae was set as 62 Mya (prior distribution: normal, mean: 62, sd: 3) [74]. (3) Based on the fossil record of *Dendrobium*, the Crown age of *Dendrobium* (the divergence time between the *Dendrobium* Asian clade and the Australian clade) was set at 21 Mya (prior distribution: normal, mean: 21, sd: 5) [25, 75]. Three independent MCMC were performed to check convergence, each with 10,000,000 generations. LogCombiner was used to integrate the data results of three independent runs and remove the top 10% of unstable data.

### Structural variation analysis of *Bulbophyllum* cp genomes

The cp genome sequences of 27 *Bulbophyllum* orchids and *D. huoshanense* were aligned using MAFFT 7.220 [64]. The InDels of each *Bulbophyllum* cp genome were counted, with *D. huoshanense* as reference. Then, the common InDels of 27 *Bulbophyllum* cp genomes were counted to calculate the InDels occurrence rates of AN, SA and *Bulbophyllum* branches. The above common InDels were excluded to calculate the recent occurrence rates of InDels in each *Bulbophyllum* cp genome.

### Nucleotide substitutions rates and positive selection analysis of protein-coding genes

The molecular evolution of 63 cp protein-coding genes were investigated from 27 *Bulbophyllum* orchids and *D. huoshanense*. The values of dN, dS and ω were estimated by the codonML in the PAML software (version 4.4) with reference *D. huoshanense* [76, 77]. Chloroplast protein-coding genes, whose ω values were different between two clades (T-test for Independent Samples) significantly, were screened. EasyCodeML was used to detect positively selective genes in the *Bulbophyllum* branch, AN branch and SA branch under Branch model with 5% significance level, respectively [40].

## Supplementary Information

**Additional file 1:** **Fig S1.** mVISTA analysis results of nine newly sequenced *Bulbophyllum* orchids with reference *D.* *huoshanense*. Chloroplast coding regions are indicated in blue, non-translation regions in cyan, andnon-coding regions in red.

**Additional file 2:** **Fig S2.** Distribution densities of LDRs and SSRs in 27 *Bulbophyllum* orchids and *D.* *huoshanense*. **a** Distribution density of LDRs; **b** Distribution density of SSRs.

**Additional file 3:** **Fig S3.** IR/SC junctions map of 19 AN *Bulbophyllum*, eight SA *Bulbophyllum* and* D.* *huoshanense*. Yellow represents the *rpl22* gene, blue represents the *ycf1* gene, red represents the *ndhF* pseudogene and green represents the *psbA* gene.

**Additional file 4:** **Fig S4.** The species tree constructed by BEAST2.

**Additional file 5:** **Fig S5.** Phylogenetic relationships of 27 orchids. **a** ML tree reconstructed by ITS sequences; **b** MLtree reconstructed by ITS sequences, *psbA*, *matK* and *rbcL*; **c **ML tree reconstructed by cp genomes. * represents 100 bootstrap value.

**Additional file 6:** **Table S1. **The species information of 61 angiosperms used in the phylogenetic analysis

**Additional file 7:** **Table S2.** dN and dS of 16 screened protein-coding genes in *Bulbophyllum.*

**Additional file 8:** **Table S3.** The basic information of 16 screened protein-coding genes.

**Additional file 9:** **Table S4.** The basic information of positively selective genes.

## Data Availability

All of the raw sequence reads used in this study have been deposited in NCBI (BioProject accession number: LC642719, LC642720, LC642721, LC642722, LC642723, LC642724, LC642725, LC642726 and LC703293).

## Abbreviations

Cp: Chloroplast
AN: Asian
SA: South American
InDels: Insertions and Deletions
CAM: Crassulacean Acid Metabolism
LSC: Large Single Copy
SSC: Small Single Copy
IR: Inverted Repeat
ITS: Internal Transcribed Spacer
LDRs: Longer Dispersed Repeats
SSRs: Simple Sequence Repeats
J_SA_s: SSC/IR_A_ junctions
J_SB_s: SSC/IR_B_ junctions
J_LB_s: LSC/IR_B_ junctions
J_L__A_s: LSC/IR_A_ junctions
SVs: Sequence Variabilities
mya: Million Years Ago
ML: Maximum Likelihood
