# Supplementary material for: Effects of geographic isolation on the Bulbophyllum chloroplast genomes
Source: BMC Plant Biol. 2022 Apr 19;22:201. doi: 10.1186/s12870-022-03592-y (PMC9016995; doi:10.1186/s12870-022-03592-y)
Supplement: Supplementary file 1 — Additional file 1: Fig S1. mVISTA analysis results of nine newly sequenced Bulbophyllum orchids with reference D. huoshanense. Chloroplast coding regions are indicated in blue, non-translation regions in cyan, andnon-coding regions in red. [file 12870_2022_3592_MOESM1_ESM.docx]

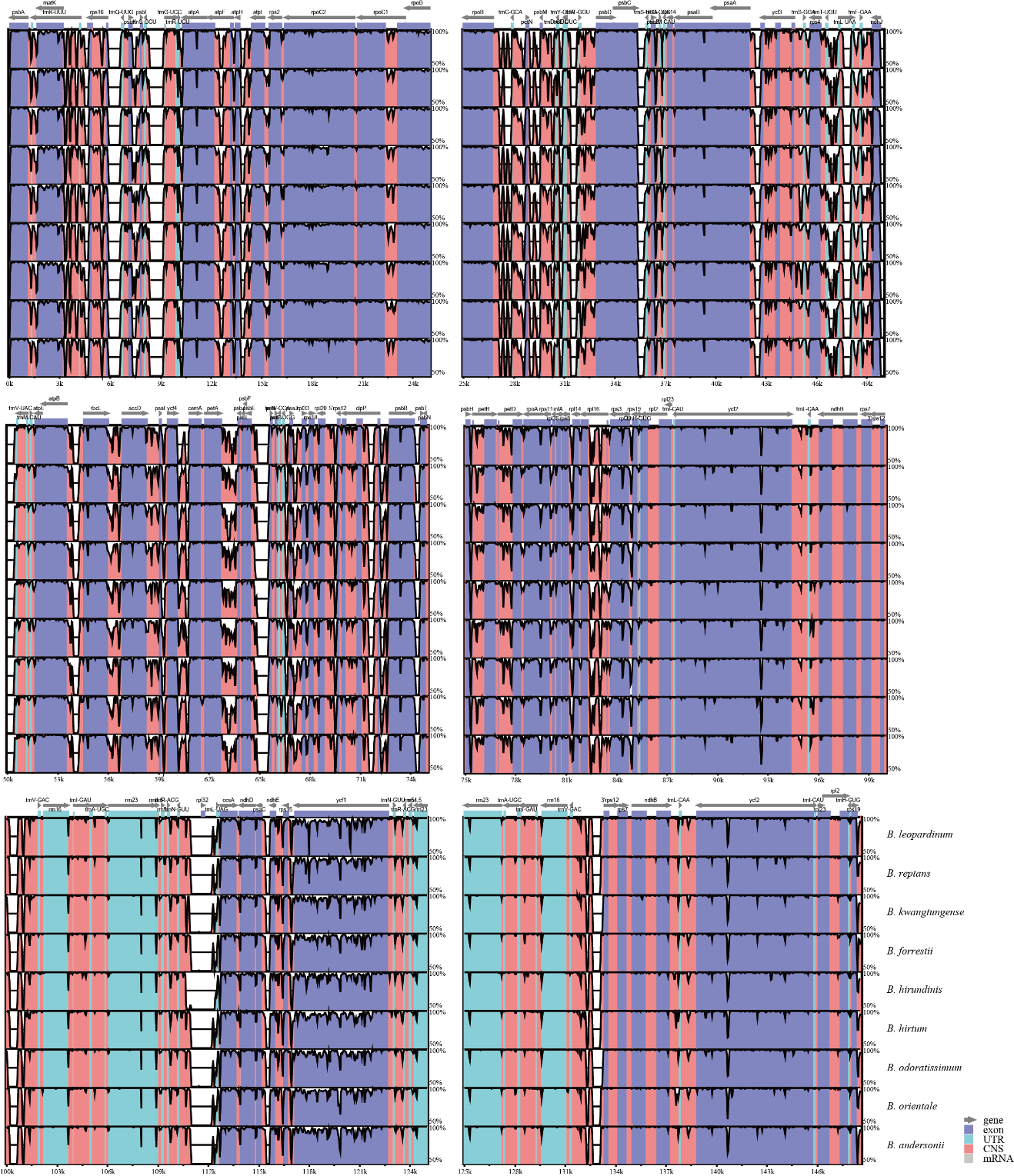


**Fig. S1** mVISTA analysis results of nine newly sequenced *Bulbophyllum* orchids with reference *D.* *huoshanense*. Chloroplast coding regions are indicated in blue, non-translation regions in cyan, and non-coding regions in red
