# Supplementary material for: Effects of geographic isolation on the Bulbophyllum chloroplast genomes
Source: BMC Plant Biol. 2022 Apr 19;22:201. doi: 10.1186/s12870-022-03592-y (PMC9016995; doi:10.1186/s12870-022-03592-y)
Supplement: Supplementary file 2 — Additional file 2: Fig S2. Distribution densities of LDRs and SSRs in 27 Bulbophyllum orchids and D. huoshanense. a Distribution density of LDRs; b Distribution density of SSRs. [file 12870_2022_3592_MOESM2_ESM.docx]

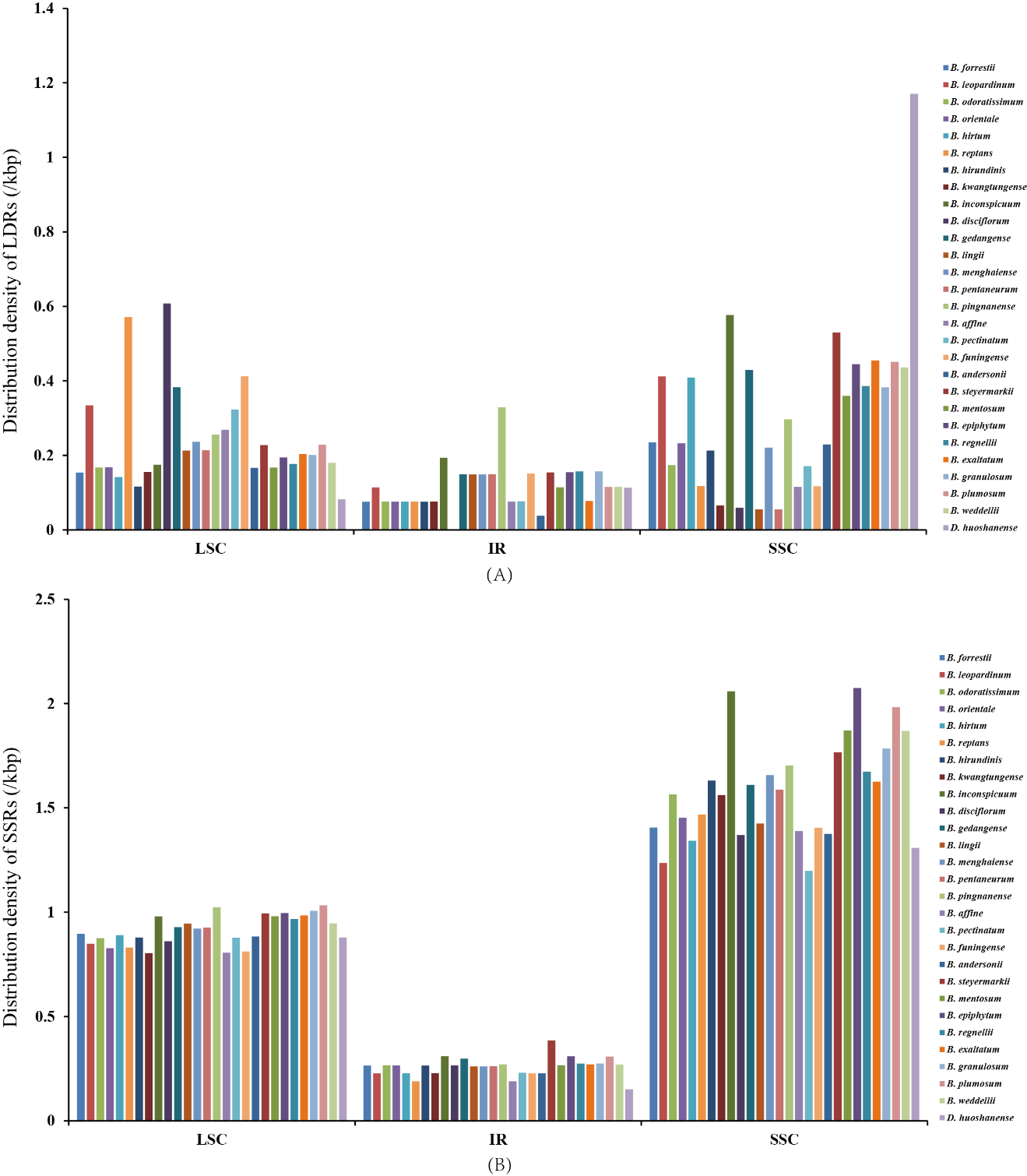


**Fig. S2** Distribution densities of LDRs and SSRs in 27 *Bulbophyllum* orchids and *D.* *huoshanense*. **a** Distribution density of LDRs; **b** Distribution density of SSRs
