# Supplementary material for: Effects of geographic isolation on the Bulbophyllum chloroplast genomes
Source: BMC Plant Biol. 2022 Apr 19;22:201. doi: 10.1186/s12870-022-03592-y (PMC9016995; doi:10.1186/s12870-022-03592-y)
Supplement: Supplementary file 3 — Additional file 3: Fig S3. IR/SC junctions map of 19 AN Bulbophyllum, eight SA Bulbophyllum and D. huoshanense. Yellow represents the rpl22 gene, blue represents the ycf1 gene, red represents the ndhF pseudogene and green represents the psbA gene. [file 12870_2022_3592_MOESM3_ESM.docx]

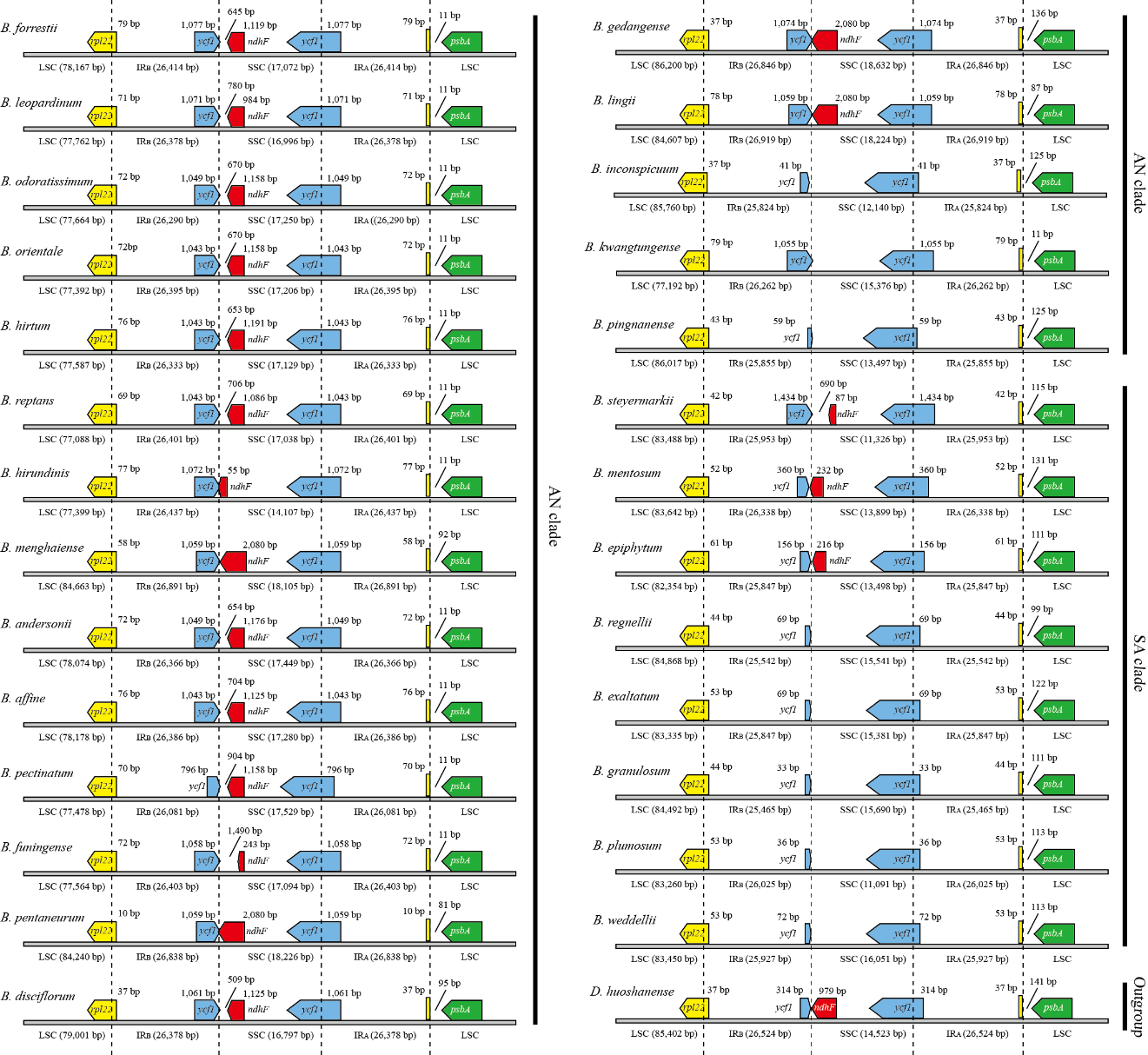


**Fig. S3** IR/SC junctions map of 19 AN *Bulbophyllum*, eight SA *Bulbophyllum* and *D.* *huoshanense*. Yellow represents the *rpl22* gene, blue represents the *ycf1* gene, red represents the *ndhF* pseudogene and green represents the *psbA* gene
