# Supplementary figures and images for: Effects of geographic isolation on the Bulbophyllum chloroplast genomes
Source: BMC Plant Biol. 2022 Apr 19;22:201. doi: 10.1186/s12870-022-03592-y (PMC9016995; doi:10.1186/s12870-022-03592-y)

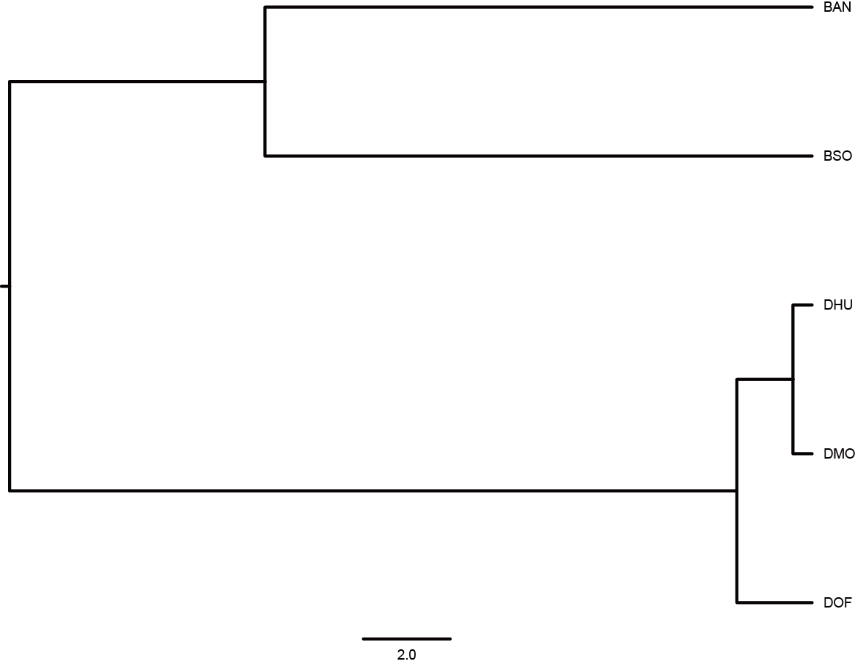


**Fig. S4** The species tree constructed by BEAST2

Supplement: Supplementary file 4 — Additional file 4: Fig S4. The species tree constructed by BEAST2. [file 12870_2022_3592_MOESM4_ESM.docx]
