# Supplementary material for: Effects of geographic isolation on the Bulbophyllum chloroplast genomes
Source: BMC Plant Biol. 2022 Apr 19;22:201. doi: 10.1186/s12870-022-03592-y (PMC9016995; doi:10.1186/s12870-022-03592-y)
Supplement: Supplementary file 5 — Additional file 5: Fig S5. Phylogenetic relationships of 27 orchids. a ML tree reconstructed by ITS sequences; b MLtree reconstructed by ITS sequences, psbA, matK and rbcL; c ML tree reconstructed by cp genomes. * represents 100 bootstrap value. [file 12870_2022_3592_MOESM5_ESM.docx]

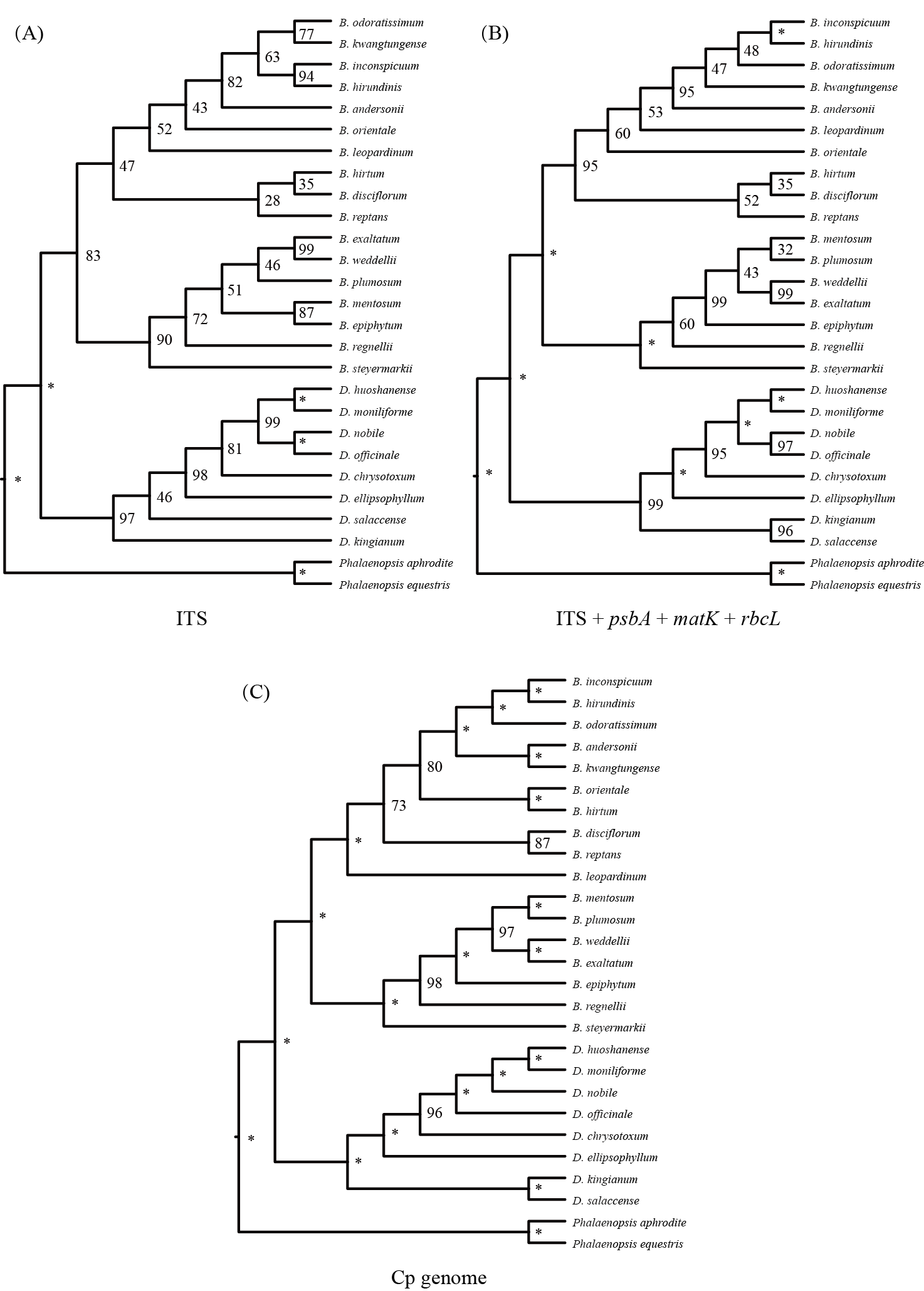


**Fig. S5** Phylogenetic relationships of 27 orchids. **a** ML tree reconstructed by ITS sequences; **b** ML tree reconstructed by ITS sequences, *psbA*, *matK* and *rbcL*; **c** ML tree reconstructed by cp genomes. * represents 100 bootstrap value
