# Supplementary material for: Effects of geographic isolation on the Bulbophyllum chloroplast genomes
Source: BMC Plant Biol. 2022 Apr 19;22:201. doi: 10.1186/s12870-022-03592-y (PMC9016995; doi:10.1186/s12870-022-03592-y)
Supplement: Supplementary file 6 — Additional file 6: Table S1. The species information of 61 angiosperms used in the phylogenetic analysis [file 12870_2022_3592_MOESM6_ESM.docx]

**Table S1** The species information of 61 angiosperms used in the phylogenetic analysis

| no. | Class | Order | Family | Species | Accession no. |
| --- | --- | --- | --- | --- | --- |
| 1 | Monocotyledoneae | Asparagales | Orchidaceae | *Goodyera fumata* | NC_026773 |
| 2 | Monocotyledoneae | Asparagales | Orchidaceae | *Goodyera schlechtendaliana* | LC085346 |
| 3 | Monocotyledoneae | Asparagales | Orchidaceae | *Goodyera procera* | NC_029363 |
| 4 | Monocotyledoneae | Asparagales | Orchidaceae | *Anoectochilus emeiensis* | NC_033895 |
| 5 | Monocotyledoneae | Asparagales | Orchidaceae | *Ludisia discolor* | NC_030540 |
| 6 | Monocotyledoneae | Asparagales | Orchidaceae | *Habenaria pantlingiana* | NC_026775 |
| 7 | Monocotyledoneae | Asparagales | Orchidaceae | *Bulbophyllum exaltatum* | NC_048480 |
| 8 | Monocotyledoneae | Asparagales | Orchidaceae | *Bulbophyllum weddellii* | NC_048485 |
| 9 | Monocotyledoneae | Asparagales | Orchidaceae | *Bulbophyllum epiphytum* | NC_048486 |
| 10 | Monocotyledoneae | Asparagales | Orchidaceae | *Bulbophyllum mentosum* | NC_048482 |
| 11 | Monocotyledoneae | Asparagales | Orchidaceae | *Bulbophyllum plumosum* | NC_048479 |
| 12 | Monocotyledoneae | Asparagales | Orchidaceae | *Bulbophyllum granulosum* | NC_048481 |
| 13 | Monocotyledoneae | Asparagales | Orchidaceae | *Bulbophyllum regnellii* | NC_048483 |
| 14 | Monocotyledoneae | Asparagales | Orchidaceae | *Bulbophyllum steyermarkii* | NC_048484 |
| 15 | Monocotyledoneae | Asparagales | Orchidaceae | *Bulbophyllum forrestii* | LC642719 |
| 16 | Monocotyledoneae | Asparagales | Orchidaceae | *Bulbophyllum odoratissimum* | LC642720 |
| 17 | Monocotyledoneae | Asparagales | Orchidaceae | *Bulbophyllum hirundinis* | LC642721 |
| 18 | Monocotyledoneae | Asparagales | Orchidaceae | *Bulbophyllum inconspicuum* | NC_046811 |
| 19 | Monocotyledoneae | Asparagales | Orchidaceae | *Bulbophyllum kwangtungense* | LC642722 |
| 20 | Monocotyledoneae | Asparagales | Orchidaceae | *Bulbophyllum leopardinum* | LC642723 |
| 21 | Monocotyledoneae | Asparagales | Orchidaceae | *Bulbophyllum hirtum* | LC642724 |
| 22 | Monocotyledoneae | Asparagales | Orchidaceae | *Bulbophyllum orientale* | LC642725 |
| 23 | Monocotyledoneae | Asparagales | Orchidaceae | *Bulbophyllum reptans* | LC642726 |
| 24 | Monocotyledoneae | Asparagales | Orchidaceae | *Bulbophyllum disciflorum* | LC498826 |
| 25 | Monocotyledoneae | Asparagales | Orchidaceae | *Bulbophyllum gedangense* | MW161053 |
| 26 | Monocotyledoneae | Asparagales | Orchidaceae | *Bulbophyllum lingii* | MW161052 |
| 27 | Monocotyledoneae | Asparagales | Orchidaceae | *Bulbophyllum menghaiense* | MW161050 |
| 28 | Monocotyledoneae | Asparagales | Orchidaceae | *Bulbophyllum pentaneurum* | MW161051 |
| 29 | Monocotyledoneae | Asparagales | Orchidaceae | *Bulbophyllum pingnanense* | MW822749 |
| 30 | Monocotyledoneae | Asparagales | Orchidaceae | *Bulbophyllum affine* | LC556091 |
| 31 | Monocotyledoneae | Asparagales | Orchidaceae | *Bulbophyllum pectinatum* | LC556092 |
| 32 | Monocotyledoneae | Asparagales | Orchidaceae | *Bulbophyllum funingense* | LC556093 |
| 33 | Monocotyledoneae | Asparagales | Orchidaceae | *Bulbophyllum andersonii* | LC703293 |
| 34 | Monocotyledoneae | Asparagales | Orchidaceae | *Dendrobium huoshanense* | NC_028430 |
| 35 | Monocotyledoneae | Asparagales | Orchidaceae | *Dendrobium moniliforme* | NC_035154 |
| 36 | Monocotyledoneae | Asparagales | Orchidaceae | *Dendrobium nobile* | LC011413 |
| 37 | Monocotyledoneae | Asparagales | Orchidaceae | *Dendrobium officinale* | NC_024019 |
| 38 | Monocotyledoneae | Asparagales | Orchidaceae | *Dendrobium chrysotoxum* | LC193517 |
| 39 | Monocotyledoneae | Asparagales | Orchidaceae | *Dendrobium ellipsophyllum* | NC_035340 |
| 40 | Monocotyledoneae | Asparagales | Orchidaceae | *Dendrobium kingianum* | LC331062 |
| 41 | Monocotyledoneae | Asparagales | Orchidaceae | *Dendrobium salaccense* | NC_035332 |
| 42 | Monocotyledoneae | Asparagales | Orchidaceae | *Phalaenopsis aphrodite* | NC_007499 |
| 43 | Monocotyledoneae | Asparagales | Orchidaceae | *Phalaenopsis equestris* | NC_017609 |
| 44 | Monocotyledoneae | Asparagales | Orchidaceae | *Bletilla ochracea* | NC_029483 |
| 45 | Monocotyledoneae | Asparagales | Orchidaceae | *Bletilla striata* | NC_028422 |
| 46 | Monocotyledoneae | Asparagales | Orchidaceae | *Sobralia aff. bouchei* | NC_028209 |
| 47 | Monocotyledoneae | Asparagales | Orchidaceae | *Sobralia callosa* | NC_028147 |
| 48 | Monocotyledoneae | Asparagales | Orchidaceae | *Elleanthus sodiroi* | NC_027266 |
| 49 | Monocotyledoneae | Asparagales | Orchidaceae | *Paphiopedilum armeniacum* | LC085347 |
| 50 | Monocotyledoneae | Asparagales | Orchidaceae | *Paphiopedilum niveum* | NC_026776 |
| 51 | Monocotyledoneae | Asparagales | Orchidaceae | *Phragmipedium longifolium* | NC_028149 |
| 52 | Monocotyledoneae | Asparagales | Orchidaceae | *Neuwiedia zollingeri* | LC199503 |
| 53 | Monocotyledoneae | Liliales | Liliaceae | *Fritillaria taipaiensis* | NC_023247 |
| 54 | Monocotyledoneae | Liliales | Liliaceae | *Lilium longiflorum* | KC968977 |
| 55 | Monocotyledoneae | Liliales | Smilacaceae | *Smilax china* | HM536959 |
| 56 | Monocotyledoneae | Liliales | Alstroemeriaceae | *Alstroemeria aurea* | KC968976 |
| 57 | Monocotyledoneae | Alismatales | Araceae | *Lemna minor* | NC_010109 |
| 58 | Monocotyledoneae | Alismatales | Araceae | *Wolffia australiana* | JN160605 |
| 59 | Monocotyledoneae | Alismatales | Araceae | *Spirodela polyrhiza* | NC_015891 |
| 60 | Monocotyledoneae | Alismatales | Araceae | *Colocasia esculenta* | JN105689 |
| 61 | Dicotyledoneae | Brassicales | Brassicaceae | *Arabidopsis thaliana* | NC_000932 |
