# Supplementary material for: Effects of geographic isolation on the Bulbophyllum chloroplast genomes
Source: BMC Plant Biol. 2022 Apr 19;22:201. doi: 10.1186/s12870-022-03592-y (PMC9016995; doi:10.1186/s12870-022-03592-y)
Supplement: Supplementary file 7 — Additional file 7: Table S2. dN and dS of 16 screened protein-coding genes in Bulbophyllum. [file 12870_2022_3592_MOESM7_ESM.docx]

**Table S2** dN and dS of 16 screened protein-coding genes in *Bulbophyllum*

| Species | Genes | dN | dS |
| --- | --- | --- | --- |
| *B. disciflorum* | *atpA* | 0.0043 | 0.0425 |
|  | *atpE* | 0.0034 | 0.0103 |
|  | *cemA* | 0.0095 | 0.035 |
|  | *psbZ* | 0.0073 | 0.024 |
|  | *rpl16* | 0.0032 | 0.05 |
|  | *rpl2* | 0 | 0 |
|  | *rpl36* | 0 | 0.0336 |
|  | *rpoA* | 0.0108 | 0.0767 |
|  | *rpoC1* | 0.0084 | 0.0381 |
|  | *rps14* | 0 | 0.0116 |
|  | *rps15* | 0.0099 | 0.0309 |
|  | *rps19* | 0.0045 | 0 |
|  | *rps4* | 0.0045 | 0.013 |
|  | *rps8* | 0.0168 | 0.0656 |
|  | *ycf3* | 0.0077 | 0.0295 |
|  | *ycf4* | 0.0054 | 0.0309 |
| *B. epiphytum* | *atpA* | 0.0087 | 0.0648 |
|  | *atpE* | 0.0068 | 0.0329 |
|  | *cemA* | 0.0088 | 0.0286 |
|  | *psbZ* | 0 | 0.0473 |
|  | *rpl16* | 0.0096 | 0.0258 |
|  | *rpl2* | 0.0032 | 0 |
|  | *rpl36* | 0.0129 | 0.034 |
|  | *rpoA* | 0.0126 | 0.1058 |
|  | *rpoC1* | 0.0114 | 0.0304 |
|  | *rps14* | 0 | 0.0195 |
|  | *rps15* | 0.019 | 0.0881 |
|  | *rps19* | 0.0045 | 0.0198 |
|  | *rps4* | 0.009 | 0.0138 |
|  | *rps8* | 0.0236 | 0.0516 |
|  | *ycf3* | 0.0105 | 0.038 |
|  | *ycf4* | 0.0103 | 0.0492 |
| *B. exaltatum* | *atpA* | 0.006 | 0.0543 |
|  | *atpE* | 0.0034 | 0.021 |
|  | *cemA* | 0.0088 | 0.019 |
|  | *psbZ* | 0 | 0.0473 |
|  | *rpl16* | 0.0064 | 0.0256 |
|  | *rpl2* | 0.0032 | 0 |
|  | *rpl36* | 0.0129 | 0.034 |
|  | *rpoA* | 0.0083 | 0.1083 |
|  | *rpoC1* | 0.0082 | 0.0379 |
|  | *rps14* | 0 | 0.0346 |
|  | *rps15* | 0.0045 | 0.0541 |
|  | *rps19* | 0.0047 | 0.0169 |
|  | *rps4* | 0.0093 | 0.0061 |
|  | *rps8* | 0.027 | 0.0667 |
|  | *ycf3* | 0.0129 | 0.0302 |
|  | *ycf4* | 0.0109 | 0.0247 |
| *B. forrestii* | *atpA* | 0.0043 | 0.0655 |
|  | *atpE* | 0.0034 | 0.0103 |
|  | *cemA* | 0.004 | 0.03 |
|  | *psbZ* | 0.0073 | 0.024 |
|  | *rpl16* | 0.0064 | 0.0388 |
|  | *rpl2* | 0 | 0.009 |
|  | *rpl36* | 0.0001 | 0.0719 |
|  | *rpoA* | 0.0134 | 0.0768 |
|  | *rpoC1* | 0.0085 | 0.0321 |
|  | *rps14* | 0.0043 | 0.0292 |
|  | *rps15* | 0.0048 | 0.0513 |
|  | *rps19* | 0.0093 | 0.0182 |
|  | *rps4* | 0.0048 | 0.0111 |
|  | *rps8* | 0.0168 | 0.0654 |
|  | *ycf3* | 0.0077 | 0.0295 |
|  | *ycf4* | 0.005 | 0.0435 |
| *B. gedangense* | *atpA* | 0.0043 | 0.062 |
|  | *atpE* | 0.0034 | 0.0103 |
|  | *cemA* | 0.0058 | 0.0321 |
|  | *psbZ* | 0.0073 | 0.024 |
|  | *rpl16* | 0.0031 | 0.0536 |
|  | *rpl2* | 0 | 0.009 |
|  | *rpl36* | 0.0001 | 0.0719 |
|  | *rpoA* | 0.0164 | 0.0817 |
|  | *rpoC1* | 0.0084 | 0.036 |
|  | *rps14* | 0.0043 | 0.0292 |
|  | *rps15* | 0.0048 | 0.0513 |
|  | *rps19* | 0.0045 | 0.0198 |
|  | *rps4* | 0.0048 | 0.0166 |
|  | *rps8* | 0.0168 | 0.0654 |
|  | *ycf3* | 0.0077 | 0.0295 |
|  | *ycf4* | 0.0074 | 0.0454 |
| *B. granulosum* | *atpA* | 0.007 | 0.0471 |
|  | *atpE* | 0.0066 | 0.0463 |
|  | *cemA* | 0.0089 | 0.0269 |
|  | *psbZ* | 0.0001 | 0.0776 |
|  | *rpl16* | 0.0064 | 0.0399 |
|  | *rpl2* | 0.0064 | 0.0001 |
|  | *rpl36* | 0.0129 | 0.034 |
|  | *rpoA* | 0.0173 | 0.0769 |
|  | *rpoC1* | 0.0082 | 0.0326 |
|  | *rps14* | 0 | 0.0345 |
|  | *rps15* | 0.0137 | 0.0575 |
|  | *rps19* | 0.0045 | 0 |
|  | *rps4* | 0.007 | 0.0184 |
|  | *rps8* | 0.0201 | 0.0658 |
|  | *ycf3* | 0.0077 | 0.0295 |
|  | *ycf4* | 0.013 | 0.0348 |
| *B. hirtum* | *atpA* | 0.0061 | 0.0542 |
|  | *atpE* | 0.0034 | 0.0211 |
|  | *cemA* | 0.0077 | 0.0132 |
|  | *psbZ* | 0.0073 | 0.024 |
|  | *rpl16* | 0.0032 | 0.0384 |
|  | *rpl2* | 0 | 0.0044 |
|  | *rpl36* | 0.0001 | 0.0719 |
|  | *rpoA* | 0.0104 | 0.0836 |
|  | *rpoC1* | 0.0079 | 0.0364 |
|  | *rps14* | 0 | 0.0116 |
|  | *rps15* | 0.0049 | 0.0498 |
|  | *rps19* | 0.0045 | 0.0198 |
|  | *rps4* | 0.0048 | 0.0111 |
|  | *rps8* | 0.0166 | 0.0527 |
|  | *ycf3* | 0.0076 | 0.0424 |
|  | *ycf4* | 0.0027 | 0.0437 |
| *B. hirundinis* | *atpA* | 0.0034 | 0.0526 |
|  | *atpE* | 0.0035 | 0.0208 |
|  | *cemA* | 0.0055 | 0.0313 |
|  | *psbZ* | 0.0072 | 0.0495 |
|  | *rpl16* | 0.0032 | 0.0527 |
|  | *rpl2* | 0 | 0.0108 |
|  | *rpl36* | 0.0001 | 0.0719 |
|  | *rpoA* | 0.0136 | 0.0799 |
|  | *rpoC1* | 0.0084 | 0.03 |
|  | *rps14* | 0.0085 | 0.032 |
|  | *rps15* | 0.0049 | 0.0667 |
|  | *rps19* | 0.0091 | 0.0197 |
|  | *rps4* | 0.0067 | 0.0209 |
|  | *rps8* | 0.0168 | 0.0654 |
|  | *ycf3* | 0.0077 | 0.0295 |
|  | *ycf4* | 0.013 | 0.0414 |
| *B. inconspicuum* | *atpA* | 0.0043 | 0.0596 |
|  | *atpE* | 0.0034 | 0.0103 |
|  | *cemA* | 0.0074 | 0.0384 |
|  | *psbZ* | 0.0072 | 0.0495 |
|  | *rpl16* | 0.0063 | 0.0403 |
|  | *rpl2* | 0 | 0.0045 |
|  | *rpl36* | 0.0001 | 0.0719 |
|  | *rpoA* | 0.0133 | 0.0864 |
|  | *rpoC1* | 0.0078 | 0.0325 |
|  | *rps14* | 0.0043 | 0.0292 |
|  | *rps15* | 0.0048 | 0.0513 |
|  | *rps19* | 0.0045 | 0.0198 |
|  | *rps4* | 0.0069 | 0.0125 |
|  | *rps8* | 0.0203 | 0.0649 |
|  | *ycf3* | 0.0077 | 0.0295 |
|  | *ycf4* | 0.0027 | 0.0378 |
| *B. kwangtungense* | *atpA* | 0.0051 | 0.0589 |
|  | *atpE* | 0.0034 | 0.0103 |
|  | *cemA* | 0.0073 | 0.016 |
|  | *psbZ* | 0.0073 | 0.024 |
|  | *rpl16* | 0.0031 | 0.0134 |
|  | *rpl2* | 0 | 0.0045 |
|  | *rpl36* | 0 | 0.0336 |
|  | *rpoA* | 0.0123 | 0.0643 |
|  | *rpoC1* | 0.0091 | 0.0347 |
|  | *rps14* | 0.0083 | 0.0179 |
|  | *rps15* | 0.0048 | 0.0357 |
|  | *rps19* | 0.0045 | 0.0198 |
|  | *rps4* | 0.0045 | 0.0131 |
|  | *rps8* | 0.0168 | 0.0516 |
|  | *ycf3* | 0.0077 | 0.0295 |
|  | *ycf4* | 0.0049 | 0.0553 |
| *B. leopardinum* | *atpA* | 0.0043 | 0.0453 |
|  | *atpE* | 0.0068 | 0.0215 |
|  | *cemA* | 0.0076 | 0.028 |
|  | *psbZ* | 0.0073 | 0.024 |
|  | *rpl16* | 0.0063 | 0.0532 |
|  | *rpl2* | 0 | 0 |
|  | *rpl36* | 0 | 0.0336 |
|  | *rpoA* | 0.0122 | 0.0723 |
|  | *rpoC1* | 0.0091 | 0.0347 |
|  | *rps14* | 0 | 0.0116 |
|  | *rps15* | 0.0146 | 0.0535 |
|  | *rps19* | 0.0045 | 0.0198 |
|  | *rps4* | 0.0048 | 0.011 |
|  | *rps8* | 0.0168 | 0.0656 |
|  | *ycf3* | 0.0077 | 0.0295 |
|  | *ycf4* | 0.0026 | 0.0417 |
| *B. lingii* | *atpA* | 0.0051 | 0.0813 |
|  | *atpE* | 0.0067 | 0.0219 |
|  | *cemA* | 0.0058 | 0.0748 |
|  | *psbZ* | 0.0074 | 0.0735 |
|  | *rpl16* | 0.0096 | 0.0686 |
|  | *rpl2* | 0 | 0.0045 |
|  | *rpl36* | 0.0127 | 0.12 |
|  | *rpoA* | 0.0279 | 0.0818 |
|  | *rpoC1* | 0.0066 | 0.0413 |
|  | *rps14* | 0 | 0.0116 |
|  | *rps15* | 0.0289 | 0.0821 |
|  | *rps19* | 0.0045 | 0.0198 |
|  | *rps4* | 0.0111 | 0.0427 |
|  | *rps8* | 0.0167 | 0.0656 |
|  | *ycf3* | 0.0105 | 0.0576 |
|  | *ycf4* | 0.0025 | 0.0745 |
| *B. mentosum* | *atpA* | 0.0073 | 0.0495 |
|  | *atpE* | 0.0034 | 0.021 |
|  | *cemA* | 0.0089 | 0.0274 |
|  | *psbZ* | 0 | 0.0473 |
|  | *rpl16* | 0.0064 | 0.0122 |
|  | *rpl2* | 0.0032 | 0 |
|  | *rpl36* | 0.0129 | 0.0728 |
|  | *rpoA* | 0.0098 | 0.1118 |
|  | *rpoC1* | 0.0082 | 0.0303 |
|  | *rps14* | 0 | 0.0345 |
|  | *rps15* | 0.0192 | 0.0392 |
|  | *rps19* | 0.009 | 0.0001 |
|  | *rps4* | 0.0093 | 0.0061 |
|  | *rps8* | 0.0199 | 0.0674 |
|  | *ycf3* | 0.0105 | 0.0285 |
|  | *ycf4* | 0.0082 | 0.0246 |
| *B. menghaiense* | *atpA* | 0.0068 | 0.0778 |
|  | *atpE* | 0.0068 | 0.0448 |
|  | *cemA* | 0.0114 | 0.0645 |
|  | *psbZ* | 0.0071 | 0.0253 |
|  | *rpl16* | 0.0096 | 0.0533 |
|  | *rpl2* | 0 | 0.0059 |
|  | *rpl36* | 0.0002 | 0.1748 |
|  | *rpoA* | 0.0263 | 0.1006 |
|  | *rpoC1* | 0.0086 | 0.044 |
|  | *rps14* | 0 | 0.0349 |
|  | *rps15* | 0.0267 | 0.0744 |
|  | *rps19* | 0 | 0 |
|  | *rps4* | 0.0116 | 0.0315 |
|  | *rps8* | 0.0167 | 0.0802 |
|  | *ycf3* | 0.0077 | 0.0624 |
|  | *ycf4* | 0.0077 | 0.0789 |
| *B. odoratissimum* | *atpA* | 0.0043 | 0.0597 |
|  | *atpE* | 0.0033 | 0.0334 |
|  | *cemA* | 0.0073 | 0.0159 |
|  | *psbZ* | 0.0074 | 0.048 |
|  | *rpl16* | 0.0064 | 0.0374 |
|  | *rpl2* | 0 | 0.009 |
|  | *rpl36* | 0.0002 | 0.1966 |
|  | *rpoA* | 0.0183 | 0.0704 |
|  | *rpoC1* | 0.0083 | 0.0443 |
|  | *rps14* | 0.0044 | 0.0424 |
|  | *rps15* | 0.0049 | 0.0652 |
|  | *rps19* | 0.0045 | 0.0198 |
|  | *rps4* | 0.0093 | 0.0061 |
|  | *rps8* | 0.0201 | 0.0802 |
|  | *ycf3* | 0.0077 | 0.0295 |
|  | *ycf4* | 0.0103 | 0.0419 |
| *B. orientale* | *atpA* | 0.0043 | 0.0388 |
|  | *atpE* | 0.0034 | 0.0103 |
|  | *cemA* | 0.0055 | 0.0151 |
|  | *psbZ* | 0.0073 | 0.024 |
|  | *rpl16* | 0.0031 | 0.0536 |
|  | *rpl2* | 0 | 0 |
|  | *rpl36* | 0 | 0.0336 |
|  | *rpoA* | 0.0091 | 0.0618 |
|  | *rpoC1* | 0.0065 | 0.0368 |
|  | *rps14* | 0 | 0.0116 |
|  | *rps15* | 0.0099 | 0.031 |
|  | *rps19* | 0.0045 | 0.0198 |
|  | *rps4* | 0.0048 | 0.0055 |
|  | *rps8* | 0.02 | 0.0535 |
|  | *ycf3* | 0.0077 | 0.0295 |
|  | *ycf4* | 0.0027 | 0.0312 |
| *B. pentaneurum* | *atpA* | 0.0077 | 0.0807 |
|  | *atpE* | 0.0067 | 0.0452 |
|  | *cemA* | 0.0079 | 0.0772 |
|  | *psbZ* | 0.0073 | 0.0779 |
|  | *rpl16* | 0.016 | 0.0551 |
|  | *rpl2* | 0.0017 | 0 |
|  | *rpl36* | 0.0127 | 0.12 |
|  | *rpoA* | 0.0232 | 0.0834 |
|  | *rpoC1* | 0.008 | 0.0436 |
|  | *rps14* | 0 | 0.0116 |
|  | *rps15* | 0.0144 | 0.0782 |
|  | *rps19* | 0 | 0 |
|  | *rps4* | 0.0068 | 0.0391 |
|  | *rps8* | 0.0168 | 0.0786 |
|  | *ycf3* | 0.0107 | 0.0371 |
|  | *ycf4* | 0.0073 | 0.0639 |
| *B. pingnanense* | *atpA* | 0.0043 | 0.053 |
|  | *atpE* | 0.0067 | 0.0111 |
|  | *cemA* | 0.0054 | 0.0253 |
|  | *psbZ* | 0.0072 | 0.0495 |
|  | *rpl16* | 0.0064 | 0.0532 |
|  | *rpl2* | 0 | 0.0108 |
|  | *rpl36* | 0.0001 | 0.0719 |
|  | *rpoA* | 0.0106 | 0.0801 |
|  | *rpoC1* | 0.0084 | 0.0308 |
|  | *rps14* | 0.0043 | 0.0292 |
|  | *rps15* | 0.0048 | 0.0513 |
|  | *rps19* | 0.0045 | 0.0198 |
|  | *rps4* | 0.0069 | 0.0125 |
|  | *rps8* | 0.0203 | 0.0652 |
|  | *ycf3* | 0.0077 | 0.0295 |
|  | *ycf4* | 0.0027 | 0.0378 |
| *B. plumosum* | *atpA* | 0.0061 | 0.0535 |
|  | *atpE* | 0.0066 | 0.0347 |
|  | *cemA* | 0.0125 | 0.0178 |
|  | *psbZ* | 0 | 0.0473 |
|  | *rpl16* | 0.0096 | 0.0251 |
|  | *rpl2* | 0.0032 | 0 |
|  | *rpl36* | 0.0262 | 0.0726 |
|  | *rpoA* | 0.0155 | 0.1107 |
|  | *rpoC1* | 0.0109 | 0.035 |
|  | *rps14* | 0 | 0.0345 |
|  | *rps15* | 0.0245 | 0.0538 |
|  | *rps19* | 0.0045 | 0 |
|  | *rps4* | 0.0093 | 0.0185 |
|  | *rps8* | 0.0168 | 0.0659 |
|  | *ycf3* | 0.0105 | 0.038 |
|  | *ycf4* | 0.0082 | 0.0309 |
| *B. regnellii* | *atpA* | 0.0061 | 0.0575 |
|  | *atpE* | 0.0066 | 0.0585 |
|  | *cemA* | 0.0124 | 0.0278 |
|  | *psbZ* | 0.0001 | 0.0776 |
|  | *rpl16* | 0.0096 | 0.0388 |
|  | *rpl2* | 0.0047 | 0 |
|  | *rpl36* | 0.0129 | 0.034 |
|  | *rpoA* | 0.0159 | 0.0941 |
|  | *rpoC1* | 0.0083 | 0.0348 |
|  | *rps14* | 0 | 0.0467 |
|  | *rps15* | 0.0137 | 0.0286 |
|  | *rps19* | 0.0045 | 0.0198 |
|  | *rps4* | 0.0094 | 0.018 |
|  | *rps8* | 0.0201 | 0.052 |
|  | *ycf3* | 0.0077 | 0.0295 |
|  | *ycf4* | 0.0105 | 0.0333 |
| *B. reptans* | *atpA* | 0.0051 | 0.0523 |
|  | *atpE* | 0.0066 | 0.0234 |
|  | *cemA* | 0.006 | 0.0177 |
|  | *psbZ* | 0.0071 | 0.0812 |
|  | *rpl16* | 0.0032 | 0.0384 |
|  | *rpl2* | 0 | 0 |
|  | *rpl36* | 0 | 0.0336 |
|  | *rpoA* | 0.01 | 0.0579 |
|  | *rpoC1* | 0.0104 | 0.0325 |
|  | *rps14* | 0 | 0.0116 |
|  | *rps15* | 0.0094 | 0.0414 |
|  | *rps19* | 0.0045 | 0.0198 |
|  | *rps4* | 0.0114 | 0.013 |
|  | *rps8* | 0.0236 | 0.052 |
|  | *ycf3* | 0.0077 | 0.0295 |
|  | *ycf4* | 0.0052 | 0.0407 |
| *B. steyermarkii* | *atpA* | 0.0061 | 0.0503 |
|  | *atpE* | 0.0034 | 0.021 |
|  | *cemA* | 0.0141 | 0.0191 |
|  | *psbZ* | 0 | 0.0473 |
|  | *rpl16* | 0.0127 | 0.0396 |
|  | *rpl2* | 0.0032 | 0 |
|  | *rpl36* | 0.0121 | 0.1281 |
|  | *rpoA* | 0.0114 | 0.0793 |
|  | *rpoC1* | 0.0108 | 0.0408 |
|  | *rps14* | 0 | 0.0345 |
|  | *rps15* | 0.0278 | 0.0551 |
|  | *rps19* | 0.0045 | 0 |
|  | *rps4* | 0.0069 | 0.0063 |
|  | *rps8* | 0.0272 | 0.0651 |
|  | *ycf3* | 0.0077 | 0.0199 |
|  | *ycf4* | 0.018 | 0.0355 |
| *B. weddellii* | *atpA* | 0.0069 | 0.0542 |
|  | *atpE* | 0.0034 | 0.021 |
|  | *cemA* | 0.0106 | 0.0281 |
|  | *psbZ* | 0 | 0.0473 |
|  | *rpl16* | 0.0064 | 0.0256 |
|  | *rpl2* | 0.0032 | 0 |
|  | *rpl36* | 0.0129 | 0.034 |
|  | *rpoA* | 0.0083 | 0.1083 |
|  | *rpoC1* | 0.009 | 0.029 |
|  | *rps14* | 0 | 0.0345 |
|  | *rps15* | 0.0189 | 0.0682 |
|  | *rps19* | 0.0045 | 0 |
|  | *rps4* | 0.0093 | 0.0122 |
|  | *rps8* | 0.0234 | 0.067 |
|  | *ycf3* | 0.0129 | 0.0302 |
|  | *ycf4* | 0.0106 | 0.0329 |
| *B. affine* | *atpA* | 0.006 | 0.0555 |
|  | *atpE* | 0.0034 | 0.0103 |
|  | *cemA* | 0.0037 | 0.0297 |
|  | *psbZ* | 0.0073 | 0.1015 |
|  | *rpl16* | 0.0032 | 0.0537 |
|  | *rpl2* | 0 | 0 |
|  | *rpl36* | 0.0131 | 0.0699 |
|  | *rpoA* | 0.0085 | 0.0649 |
|  | *rpoC1* | 0.0064 | 0.0355 |
|  | *rps14* | 0.0042 | 0.0162 |
|  | *rps15* | 0.0048 | 0.0357 |
|  | *rps19* | 0.0045 | 0 |
|  | *rps4* | 0.0048 | 0.0055 |
|  | *rps8* | 0.0202 | 0.0655 |
|  | *ycf3* | 0.0077 | 0.0295 |
|  | *ycf4* | 0.0052 | 0.0339 |
| *B. pectinatum* | *atpA* | 0.0043 | 0.0486 |
|  | *atpE* | 0.0034 | 0.0103 |
|  | *cemA* | 0.0077 | 0.0337 |
|  | *psbZ* | 0.0073 | 0.024 |
|  | *rpl16* | 0.0031 | 0.0551 |
|  | *rpl2* | 0 | 0 |
|  | *rpl36* | 0.0119 | 0.0406 |
|  | *rpoA* | 0.0168 | 0.0655 |
|  | *rpoC1* | 0.0084 | 0.033 |
|  | *rps14* | 0 | 0.0116 |
|  | *rps15* | 0.0146 | 0.0535 |
|  | *rps19* | 0.0045 | 0.0198 |
|  | *rps4* | 0.0073 | 0.011 |
|  | *rps8* | 0.0167 | 0.0517 |
|  | *ycf3* | 0.0077 | 0.0295 |
|  | *ycf4* | 0.0026 | 0.0348 |
| *B. funingense* | *atpA* | 0.006 | 0.0592 |
|  | *atpE* | 0.0034 | 0.0103 |
|  | *cemA* | 0.0073 | 0.016 |
|  | *psbZ* | 0.0073 | 0.0492 |
|  | *rpl16* | 0.0064 | 0.0247 |
|  | *rpl2* | 0 | 0.0045 |
|  | *rpl36* | 0 | 0.0336 |
|  | *rpoA* | 0.0123 | 0.0587 |
|  | *rpoC1* | 0.0085 | 0.0321 |
|  | *rps14* | 0.0042 | 0.0161 |
|  | *rps15* | 0.0048 | 0.0357 |
|  | *rps19* | 0.0045 | 0.0198 |
|  | *rps4* | 0.0024 | 0.0111 |
|  | *rps8* | 0.0238 | 0.0652 |
|  | *ycf3* | 0.0077 | 0.0295 |
|  | *ycf4* | 0.0027 | 0.0311 |
| *B. andersonii* | *atpA* | 0.0051 | 0.0588 |
|  | *atpE* | 0.0034 | 0.0103 |
|  | *cemA* | 0.0074 | 0.0222 |
|  | *psbZ* | 0.0073 | 0.024 |
|  | *rpl16* | 0.0064 | 0.0256 |
|  | *rpl2* | 0 | 0.0045 |
|  | *rpl36* | 0 | 0.0336 |
|  | *rpoA* | 0.0122 | 0.0783 |
|  | *rpoC1* | 0.0077 | 0.0356 |
|  | *rps14* | 0.0042 | 0.0161 |
|  | *rps15* | 0.0048 | 0.0357 |
|  | *rps19* | 0.0045 | 0.0198 |
|  | *rps4* | 0.0024 | 0.0111 |
|  | *rps8* | 0.0166 | 0.08 |
|  | *ycf3* | 0.0077 | 0.0295 |
|  | *ycf4* | 0.0027 | 0.0311 |
