# Supplementary material for: Effects of geographic isolation on the Bulbophyllum chloroplast genomes
Source: BMC Plant Biol. 2022 Apr 19;22:201. doi: 10.1186/s12870-022-03592-y (PMC9016995; doi:10.1186/s12870-022-03592-y)
Supplement: Supplementary file 8 — Additional file 8: Table S3. The basic information of 16 screened protein-coding genes. [file 12870_2022_3592_MOESM8_ESM.docx]

**Table S3** The basic information of 16 screened protein-coding genes

| Genes | Regions | Categories for genes |
| --- | --- | --- |
| *atpA* | LSC | Photosynthesis genes |
| *atpE* | LSC | Photosynthesis genes |
| *psbZ* | LSC | Photosynthesis genes |
| *rpl16* | LSC | Self-replication genes |
| *rpl36* | LSC | Self-replication genes |
| *rpoA* | LSC | Self-replication genes |
| *rpoC1* | LSC | Self-replication genes |
| *rps14* | LSC | Self-replication genes |
| *rps4* | LSC | Self-replication genes |
| *rps8* | LSC | Self-replication genes |
| *cemA* | LSC | Other gene |
| *ycf3* | LSC | Genes with unknown functions |
| *ycf4* | LSC | Genes with unknown functions |
| *rps15* | SSC | Self-replication genes |
| *rpl2* | IR | Self-replication genes |
| *rps19* | IR | Self-replication genes |
