# Supplementary material for: Effects of geographic isolation on the Bulbophyllum chloroplast genomes
Source: BMC Plant Biol. 2022 Apr 19;22:201. doi: 10.1186/s12870-022-03592-y (PMC9016995; doi:10.1186/s12870-022-03592-y)
Supplement: Supplementary file 9 — Additional file 9: Table S4. The basic information of positively selective genes. [file 12870_2022_3592_MOESM9_ESM.docx]

**Table S4** The basic information of positively selective genes

| Branches | Genes | Regions | Categories for genes |
| --- | --- | --- | --- |
| *Bulbophyllum* | *psbB* | LSC | Photosynthesis genes |
| *Bulbophyllum* | *psbF* | LSC | Photosynthesis genes |
| *Bulbophyllum* | *rbcL* | LSC | Photosynthesis genes |
| *Bulbophyllum* | *rpl23* | IR | Self-replication genes |
| *Bulbophyllum* | *ycf3* | LSC | Genes with unknown functions |
| *Bulbophyllum* | *ycf2* | IR | Genes with unknown functions |
| Asian | *psbF* | LSC | Photosynthesis genes |
| Asian | *psbJ* | LSC | Photosynthesis genes |
| Asian | *psbZ* | LSC | Photosynthesis genes |
| Asian | *matK* | LSC | Other gene |
| Asian | *ycf2* | IR | Genes with unknown functions |
| South American | *rpl2* | IR | Self-replication genes |
| South American | *rpl36* | LSC | Self-replication genes |
| South American | *ycf2* | IR | Genes with unknown functions |
